# Supplementary material for: Profluorescent Fluoroquinolone-Nitroxides for Investigating Antibiotic–Bacterial Interactions
Source: Antibiotics (Basel). 2019 Mar 4;8(1):19. doi: 10.3390/antibiotics8010019 (PMC6466543; doi:10.3390/antibiotics8010019)
Supplement: Supplementary file 1 [file antibiotics-08-00019-s001.pdf]

antibiotics

Article

Profluorescent Fluoroquinolone-Nitroxides for Investigating Antibiotic–Bacterial Interactions

Anthony D. Verderosa [1,2], Rabeb Dhouib [2], Kathryn E. Fairfull-Smith [1,*] and Makrina Totsika [2,*]

Supplementary Material

Table of Contents

Methods 3

Results 3

Table S1. Measured MIC values for TEMPO, TMIO, and TEIO against Gram-positive *P. aeruginosa* and *E. coli*, and Gram-negative *S. aureus*, and *E. faecalis* 4

Figure S1. Fluorescent and brightfield overlay micrographs images of bacterial cells treated with FN **14** or FM **17** 4

^{1}H NMR spectra and ^{13}C NMR spectra 4

Figure S2. ^{1}H NMR ($CDCl_3$, 600 MHz) spectrum of Nitro-TEIOMe 4

Figure S3. ^{13}C NMR ($CDCl_3$, 150 MHz) spectrum of Nitro-TEIOMe 4

Figure S4. ^{1}H NMR ($CDCl_3$, 600 MHz) spectrum of **7** 5

Figure S5. ^{13}C NMR ($CDCl_3$, 150 MHz) spectrum of **7** 5

Figure S6. ^{1}H NMR ($CDCl_3$, 600 MHz) spectrum of **8** 6

Figure S7. ^{13}C NMR ($CDCl_3$, 150 MHz) spectrum of **8** 6

Figure S8. ^{1}H NMR ($CDCl_3$, 600 MHz) spectrum of **11** 7

Figure S9. ^{13}C NMR ($CDCl_3$, 150 MHz) spectrum of **11** 7

Figure S10. ^{1}H NMR ($CDCl_3$, 600 MHz) spectrum of **9** 8

Figure S11. ^{13}C NMR ($CDCl_3$, 150 MHz) spectrum of **9** 8

Figure S12. ^{1}H NMR ($CDCl_3$, 600 MHz) spectrum of **12** 9

Figure S13. ^{13}C NMR ($CDCl_3$, 150 MHz) spectrum of **12** 9

Figure S14. ^{1}H NMR ($CDCl_3$, 600 MHz) spectrum of **10** 10

Figure S15. ^{13}C NMR ($CDCl_3$, 150 MHz) spectrum of **10** 10

Figure S16. ^{1}H NMR ($CDCl_3$, 600 MHz) spectrum of **13** 11

Figure S17. ^{13}C NMR ($CDCl_3$, 150 MHz) spectrum of **13** 11

Figure S18. ^{1}H NMR ($CDCl_3$, 600 MHz) spectrum of **14** 12

Figure S19. ^{13}C NMR ($CDCl_3$, 150 MHz) spectrum of **14** 12

Figure S20. ^{1}H NMR ($CDCl_3$, 600 MHz) spectrum of **17** ... 13
Figure S21. ^{13}C NMR ($CDCl_3$, 150 MHz) spectrum of **17** ... 13
Figure S22. ^{1}H NMR ($CDCl_3$, 600 MHz) spectrum of **15** ... 14
Figure S23. ^{13}C NMR ($CDCl_3$, 150 MHz) spectrum of **15** ... 14
Figure S24. ^{1}H NMR ($CDCl_3$, 600 MHz) spectrum of **18** ... 15
Figure S25. ^{13}C NMR ($CDCl_3$, 150 MHz) spectrum of **18** ... 15
Figure S26. ^{1}H NMR ($CDCl_3$, 600 MHz) spectrum of **16** ... 16
Figure S27. ^{13}C NMR ($CDCl_3$, 150 MHz) spectrum of **16** ... 16
Figure S28. ^{1}H NMR ($CDCl_3$, 600 MHz) spectrum of **19** ... 17
Figure S29. ^{13}C NMR ($CDCl_3$, 150 MHz) spectrum of **19** ... 17
LCMS chromatograms and HRMS spectra ... 18
Figure S30. LCMS chromatogram and HRMS spectrum of **Nitro-TEIOMe** ... 18
Figure S31. LCMS chromatogram and HRMS spectrum of **7** ... 18
Figure S32. LCMS chromatogram and HRMS spectrum of **8** ... 19
Figure S33. LCMS chromatogram and HRMS spectrum of **9** ... 19
Figure S34. LCMS chromatogram and HRMS spectrum of **10** ... 20
Figure S35. LCMS chromatogram and HRMS spectrum of **11** ... 20
Figure S36. LCMS chromatogram and HRMS spectrum of **12** ... 21
Figure S37. LCMS chromatogram and HRMS spectrum of **13** ... 21
Figure S38. LCMS chromatogram and HRMS spectrum of **14** ... 22
Figure S39. LCMS chromatogram and HRMS spectrum of **15** ... 22
Figure S40. LCMS chromatogram and HRMS spectrum of **16** ... 23
Figure S41. LCMS chromatogram and HRMS spectrum of **17** ... 23
Figure S42. LCMS chromatogram and HRMS spectrum of **18** ... 24
Figure S43. LCMS chromatogram and HRMS spectrum of **19** ... 24

Methods

MIC susceptibility assay (in 96-well plate) for unfunctionalized nitroxides:

Nitroxides 2,2,6,6-tetramethylpiperidin-1-yloxyl (TEMPO), 1,1,3,3-tetramethylisoindolin-2-yloxyl (TMIO), and 1,1,3,3-tetraethylisoindolin-2-yloxyl (TEIO) were also subjected to MIC susceptibility assays utilising the same methodology detailed above. TEMPO, TMIO, and TEIO were tested between the concentration range of 1200 to 2 μM. MIC values (Table S1) were obtained from at least 3 biological replicates, each with at least 3 technical replicates.

Results

MIC susceptibility assay for unfunctionalized nitroxides:

Table 1. Measured MIC values for TEMPO, TMIO, and TEIO against Gram-negative *P. aeruginosa* and *E. coli*, and Gram-positive *S. aureus*, and *E. faecalis*.[a].

Compound	*P. aeruginosa* ATCC 27853 MIC (μM)	*E. coli* ATCC 25922 MIC (μM)	*S. aureus* ATCC 29213 MIC (μM)	*E. faecalis* ATCC 14933 MIC (μM)
**TEMPO**	> 1200[b]	> 1200[b]	> 1200[b]	> 1200[b]
**TMIO**	> 1200[b]	> 1200[b]	> 1200[b]	> 1200[b]
**TEIO**	> 1200[b]	> 1200[b]	> 1200[b]	> 1200[b]

[a] All MICs were determined via broth microdilution method in accordance with CLSI standard; [b] Highest concentration tested.

Fluorescence microscopy images:

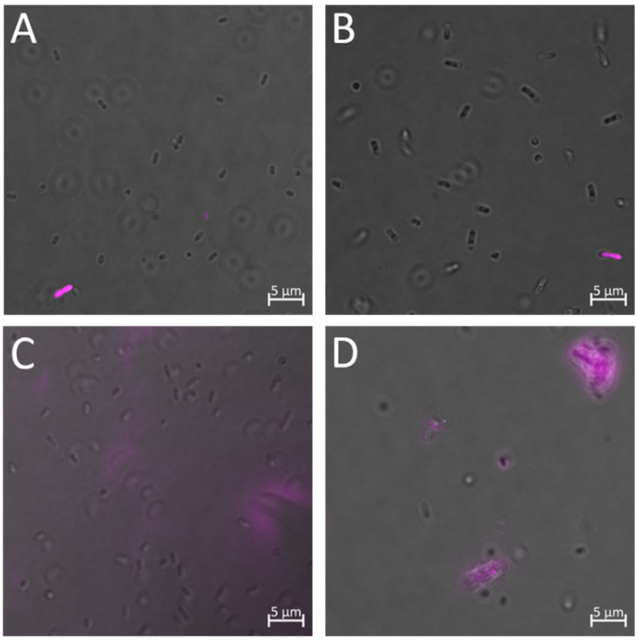

Figure S1. Fluorescent and brightfield overlay micrographs images of bacterial cells treated with FN 14 or FM 17. (A) FN 14 (150 μM) and *P. aeruginosa*; (B) FN 14 (150 μM) and *E. coli*; (C) FN 17 (150 μM) and *P. aeruginosa*; (D) FN 17 (150 μM) and *E. coli*. Scale bars are 5 μM in length.

^{1}H NMR and ^{13}C NMR Spectra

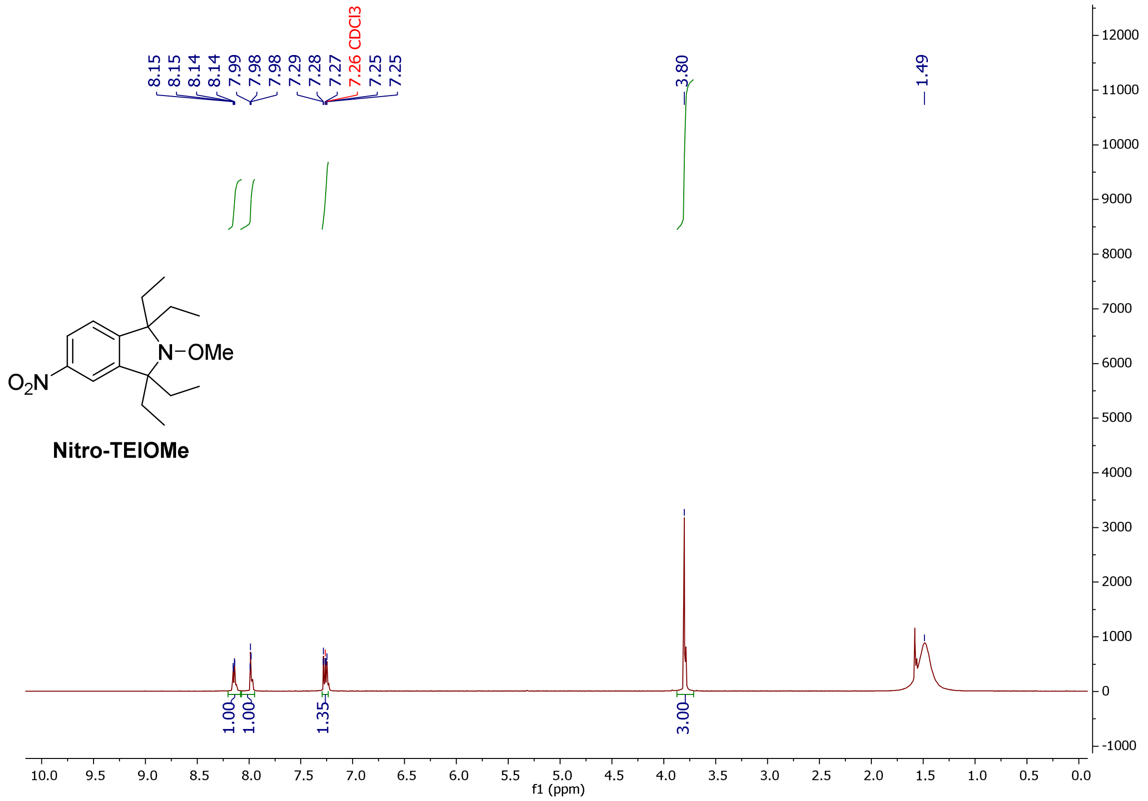

Figure S2. ^{1}H NMR ($CDCl_3$, 600 MHz) spectrum of Nitro-TEIOMe.

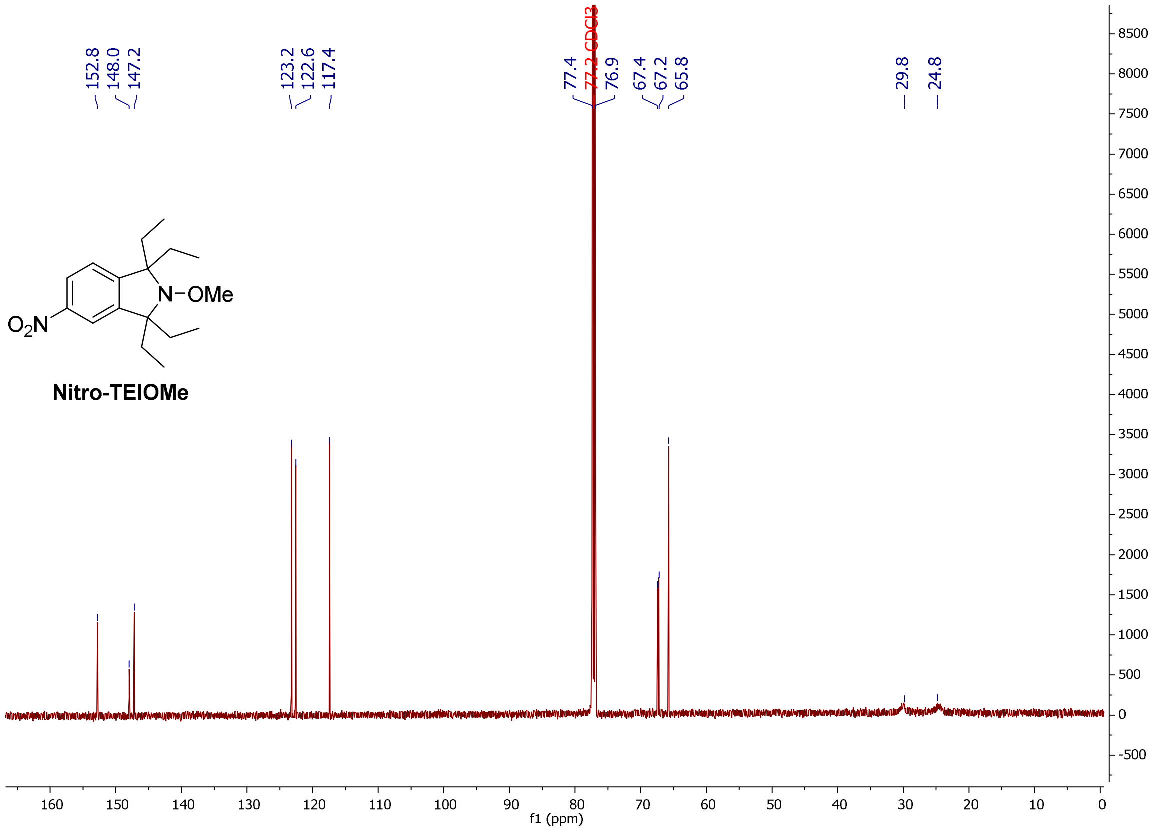

Figure S3. ^{13}C NMR ($CDCl_3$, 150 MHz) spectrum of Nitro-TEIOMe.

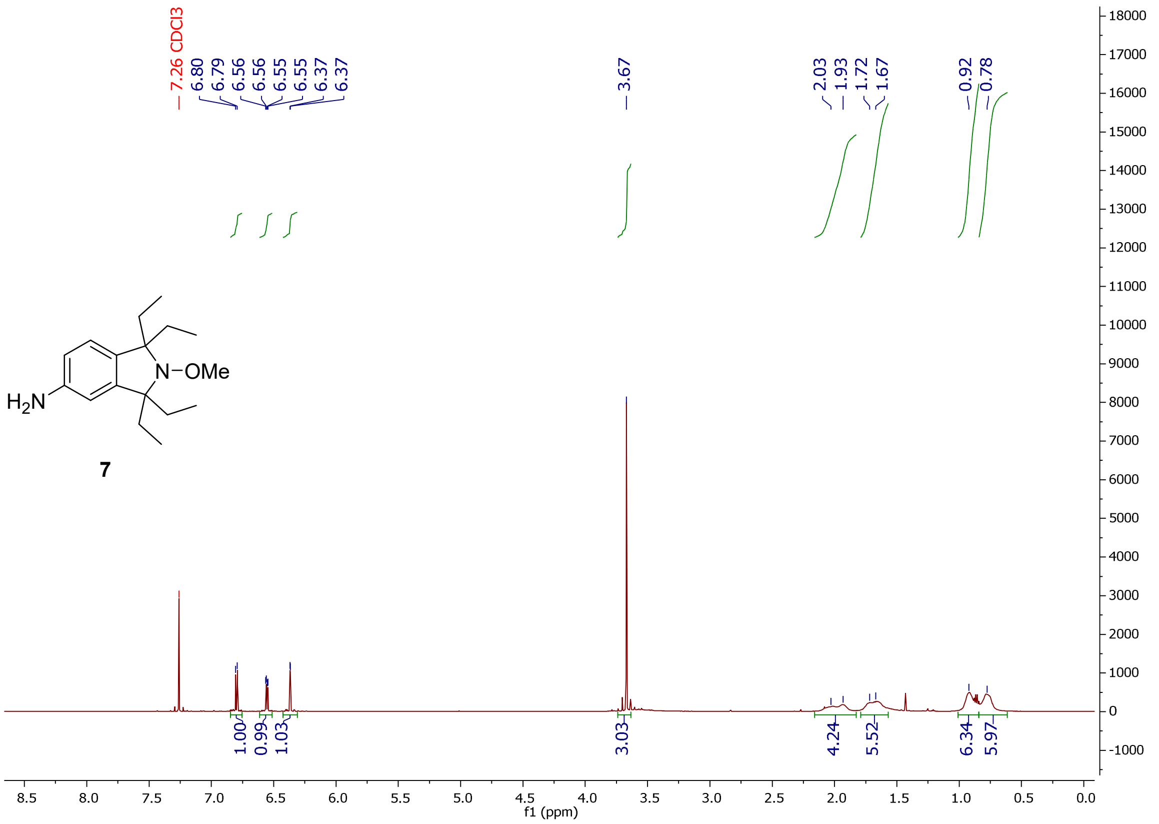

Figure S4. ^{1}H NMR ($CDCl_3$, 600 MHz) spectrum of **7**.

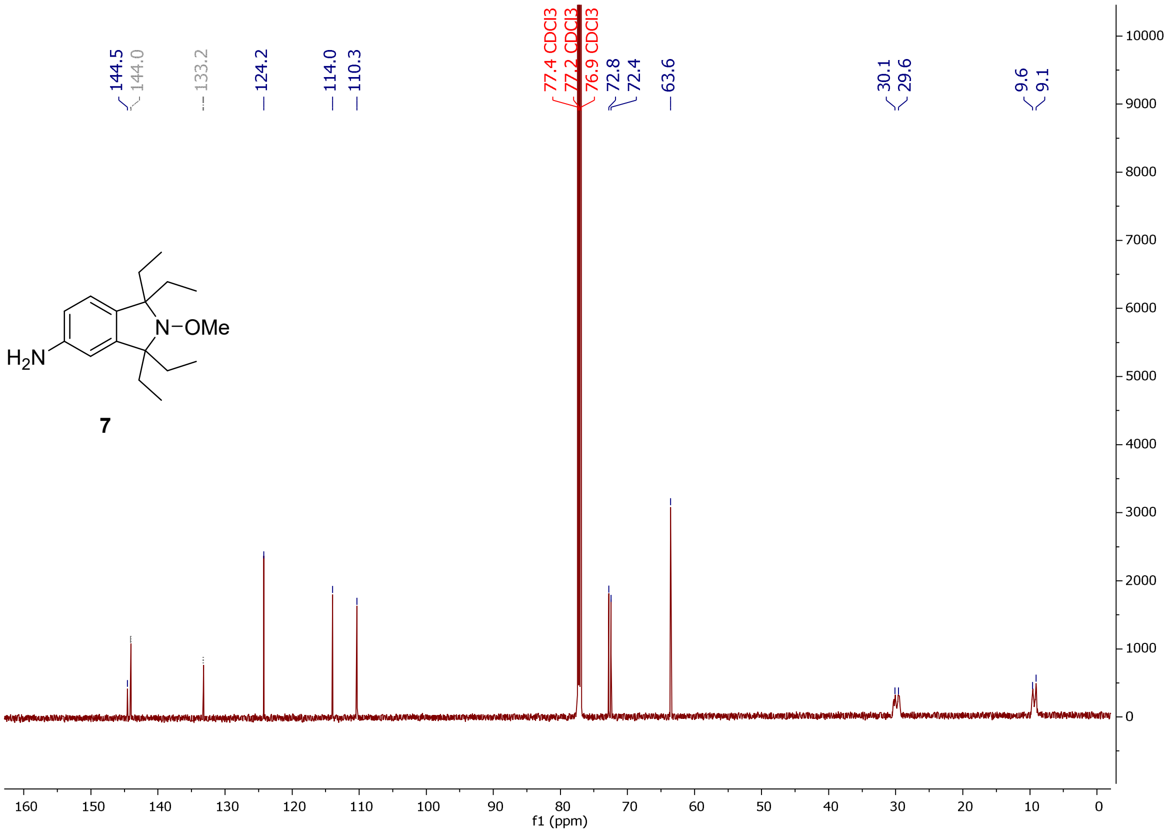

Figure S5. ^{13}C NMR ($CDCl_3$, 150 MHz) spectrum of **7**.

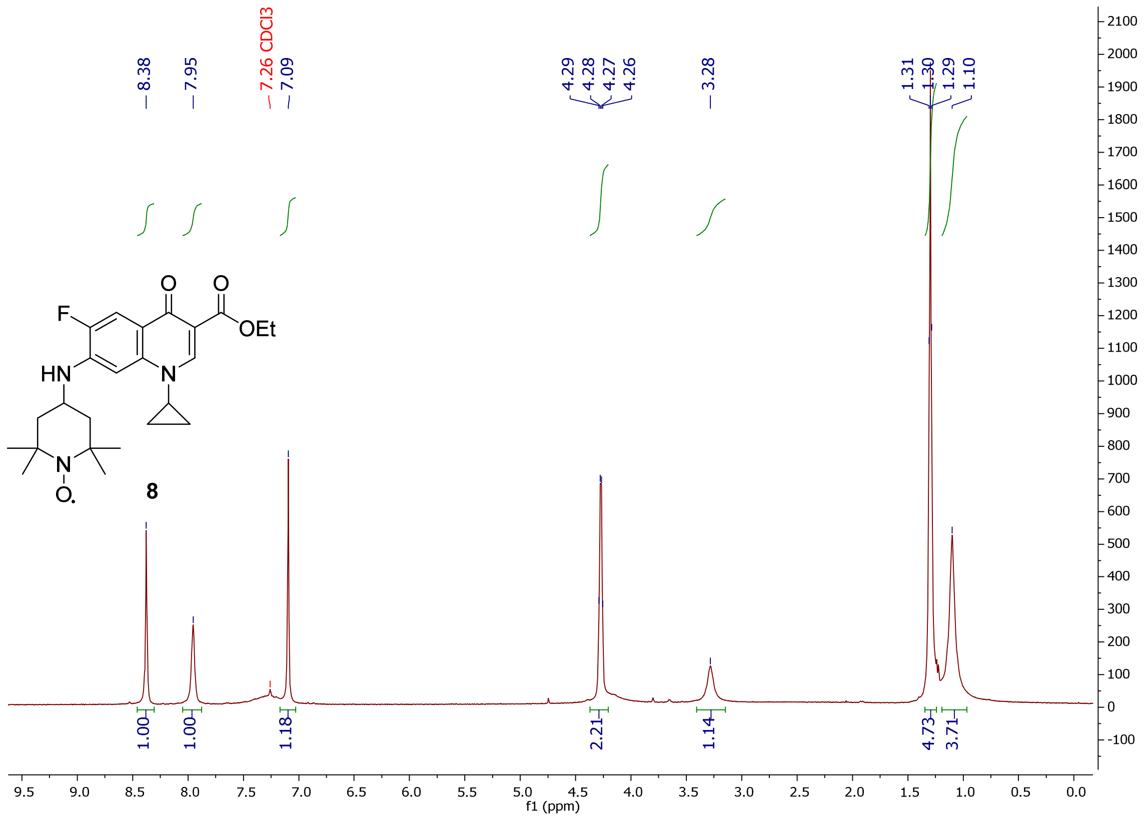

Figure 6. ^{1}H NMR ($CDCl_3$, 600 MHz) spectrum of **8**.

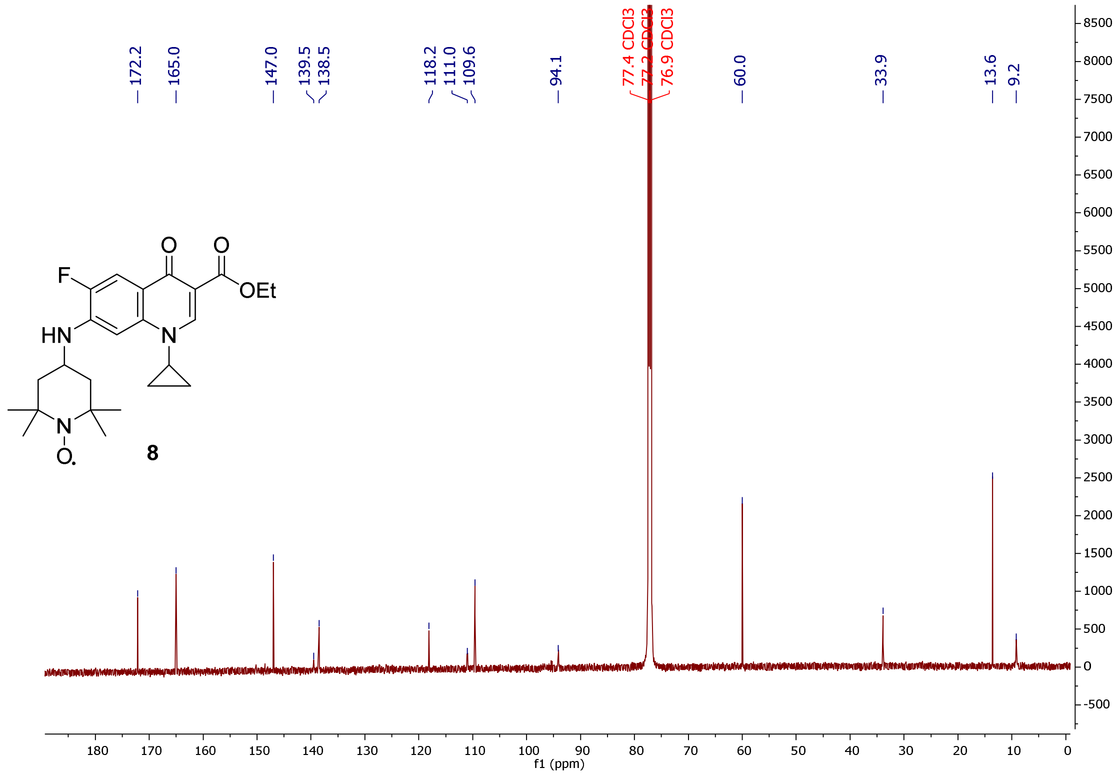

Figure S7. ^{13}C NMR ($CDCl_3$, 150 MHz) spectrum of **8**.

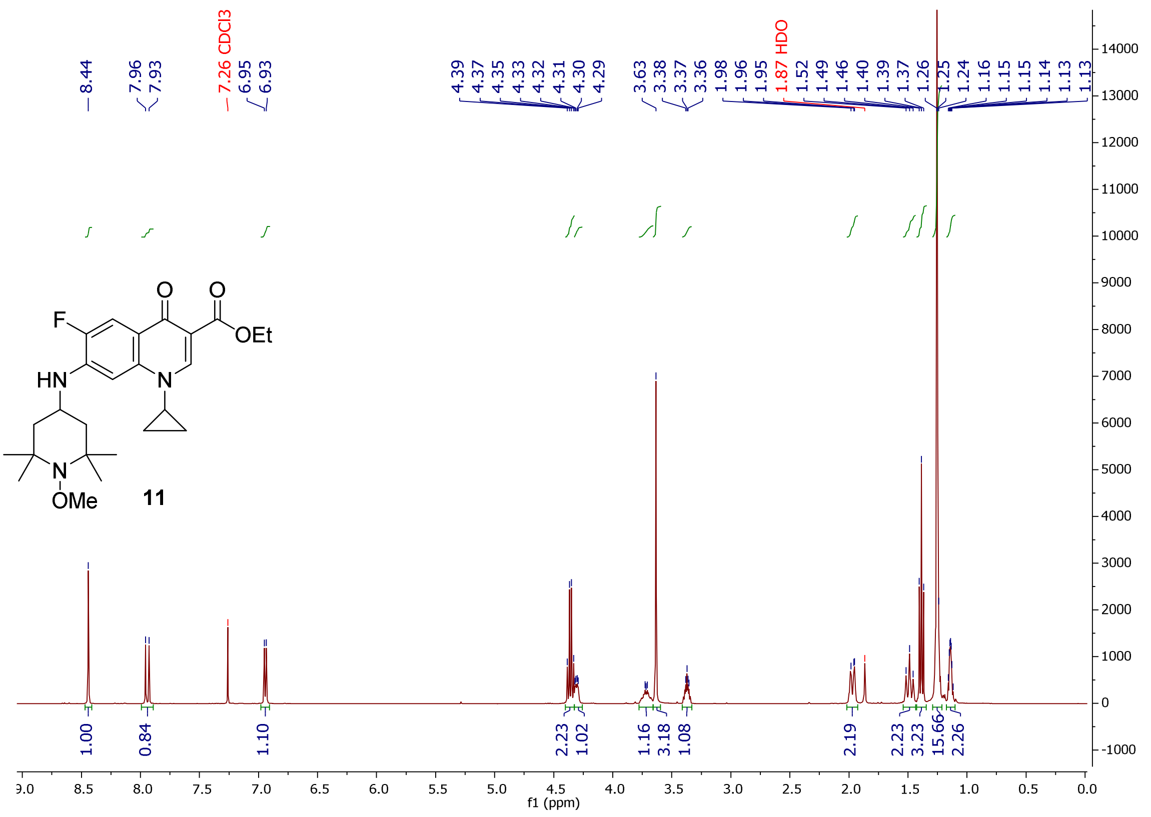

Figure 8. ^{1}H NMR ($CDCl_3$, 600 MHz) spectrum of **11**.

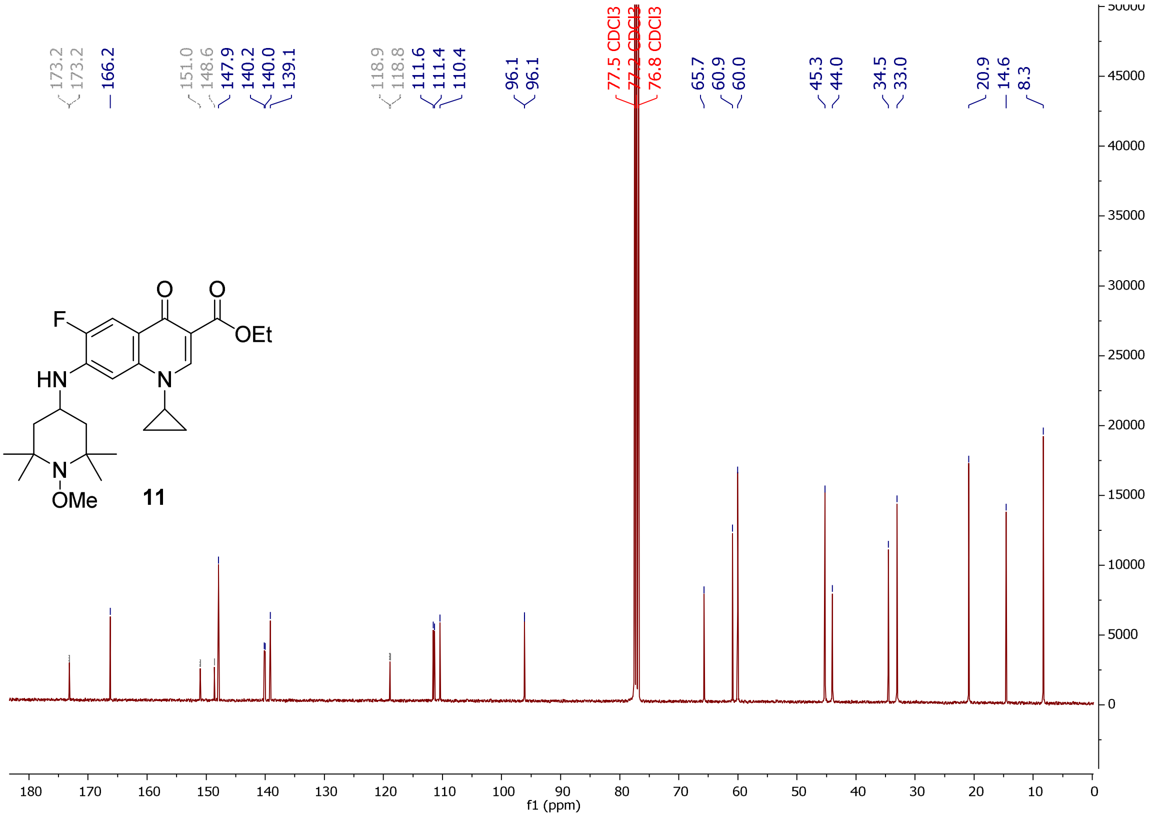

Figure S9. ^{13}C NMR ($CDCl_3$, 150 MHz) spectrum of **11.**

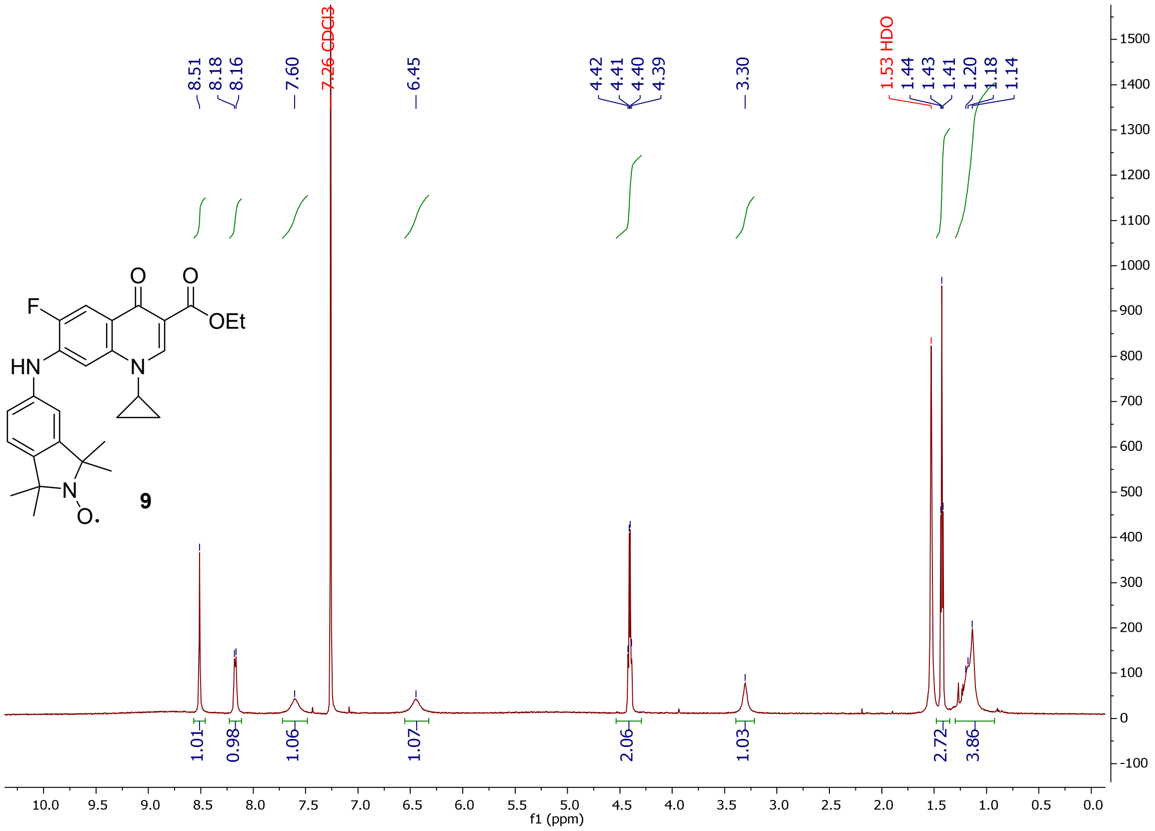

Figure S10. ^{1}H NMR ($CDCl_3$, 600 MHz) spectrum of **9**.

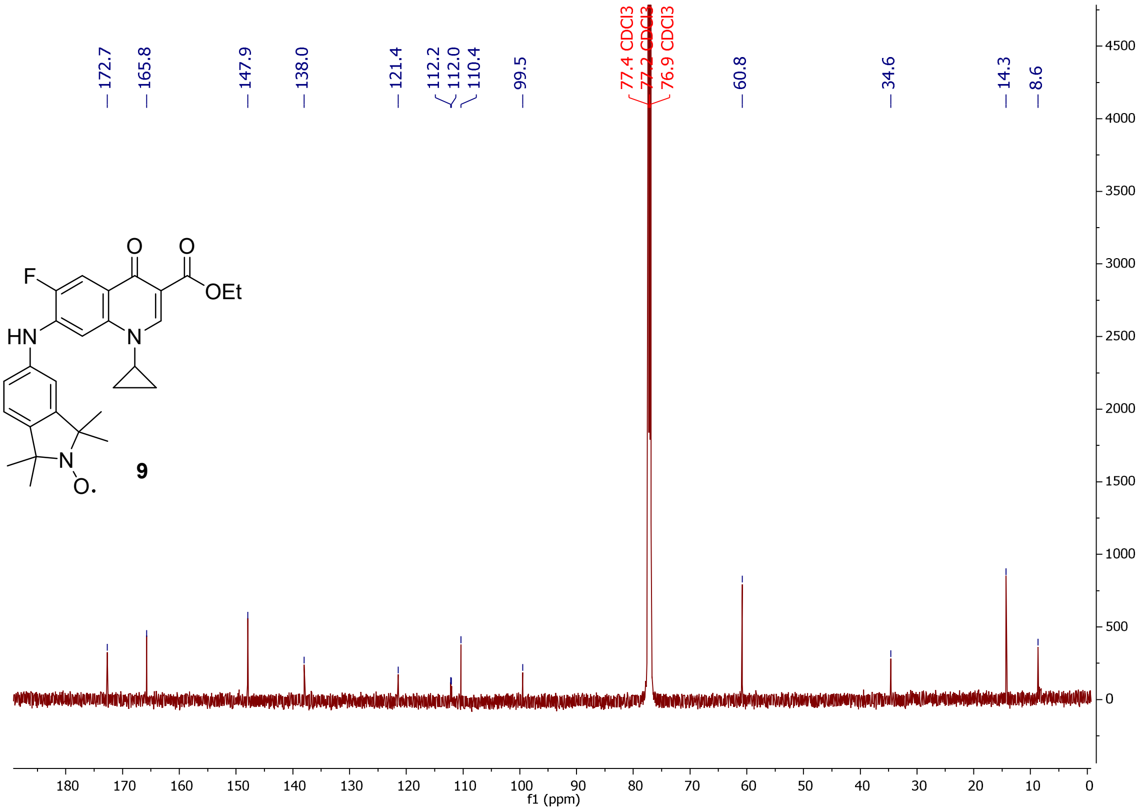

Figure S11. ^{13}C NMR ($CDCl_3$, 150 MHz) spectrum of **9**.

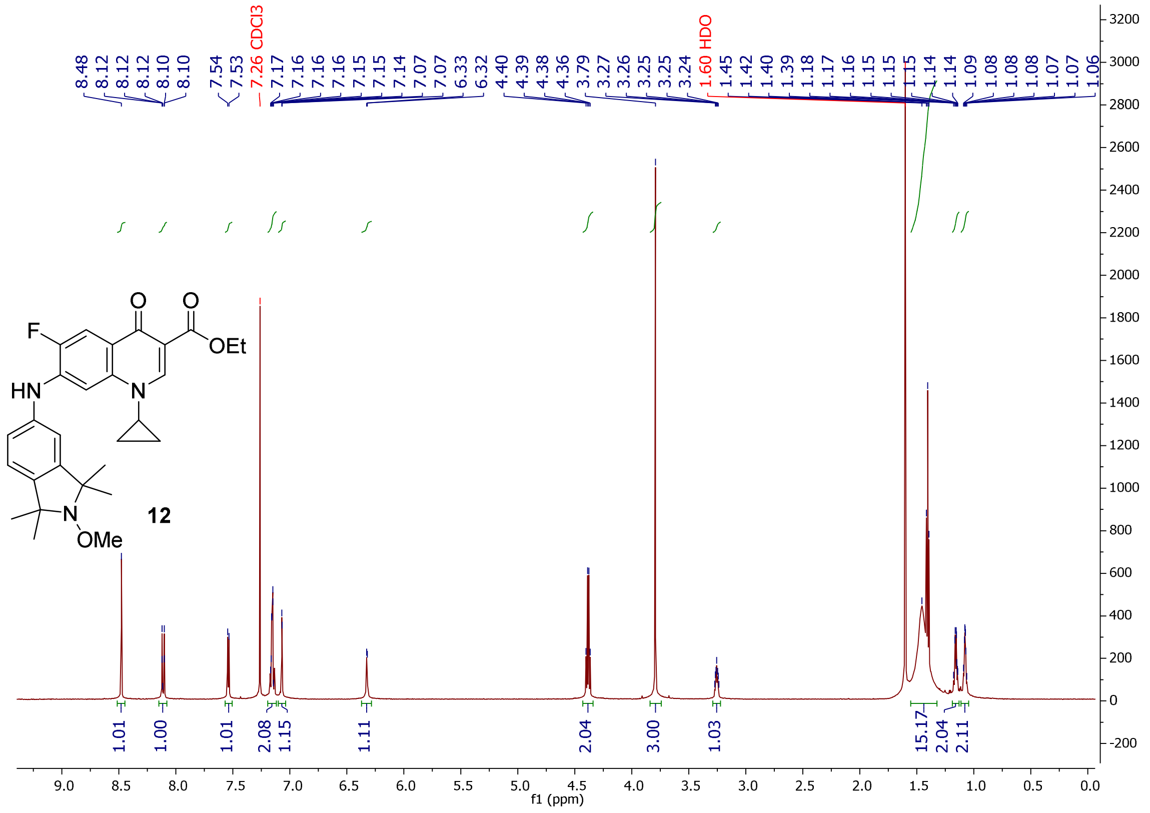

Figure S12. ^{1}H NMR ($CDCl_3$, 600 MHz) spectrum of **12**.

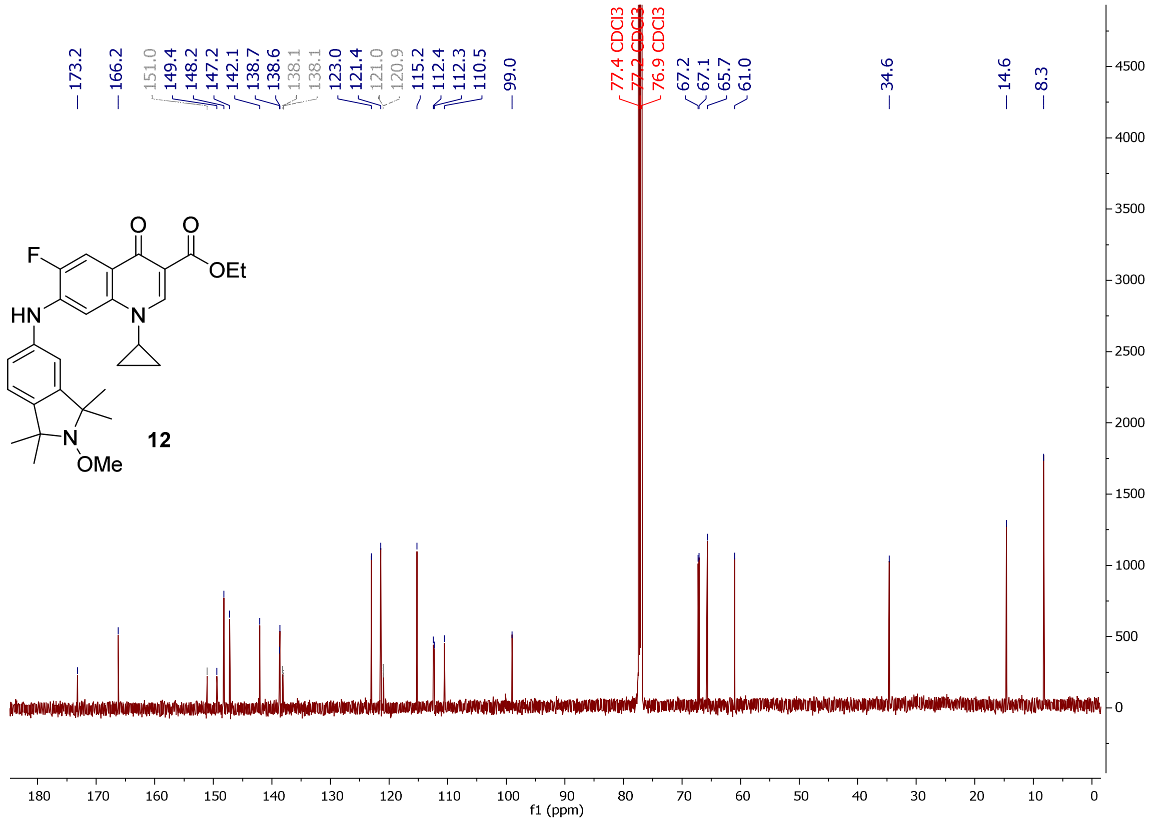

Figure S13. ^{13}C NMR ($CDCl_3$, 150 MHz) spectrum of **12**.

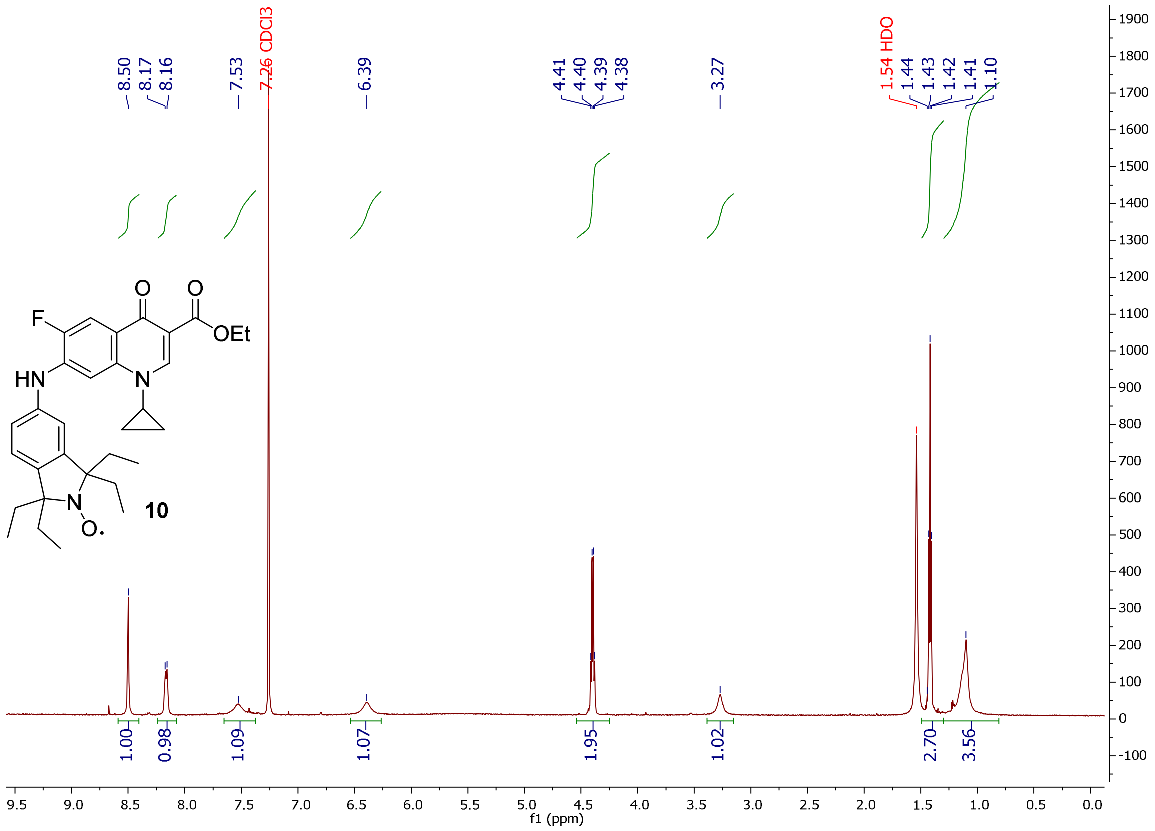

Figure S14. ^{1}H NMR ($CDCl_3$, 600 MHz) spectrum of **10**.

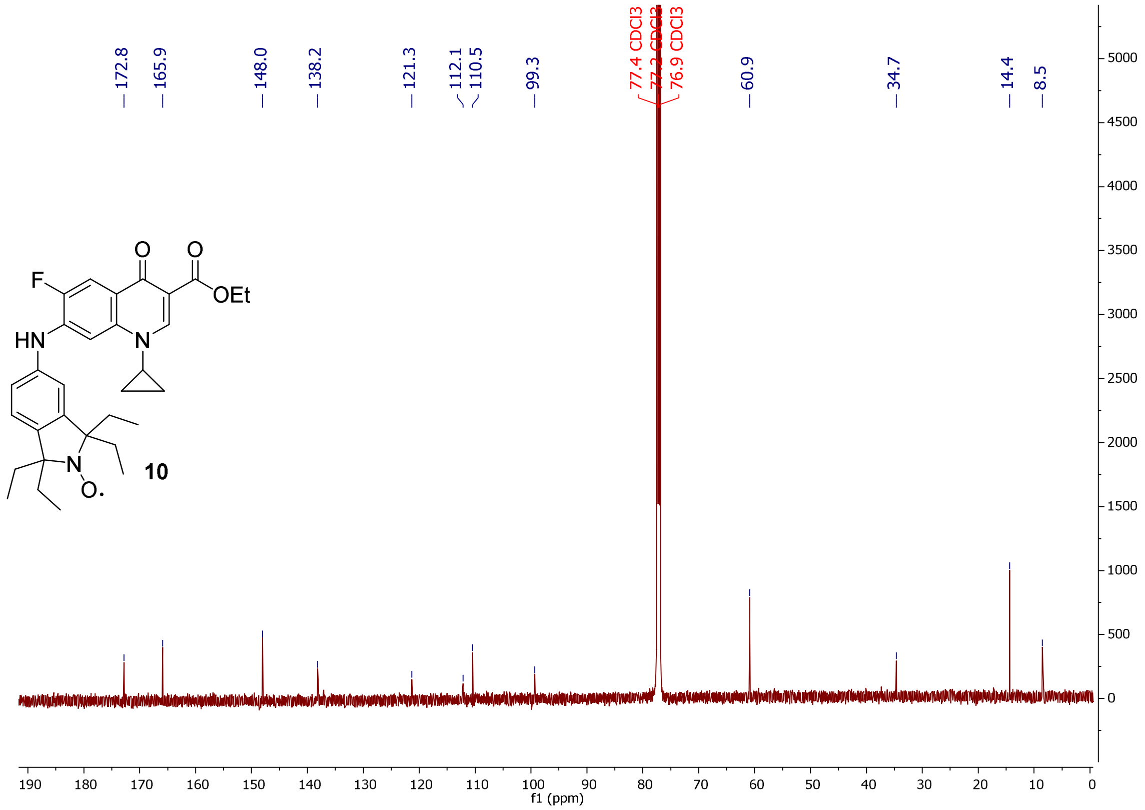

Figure S15. ^{13}C NMR ($CDCl_3$, 150 MHz) spectrum of **10**.

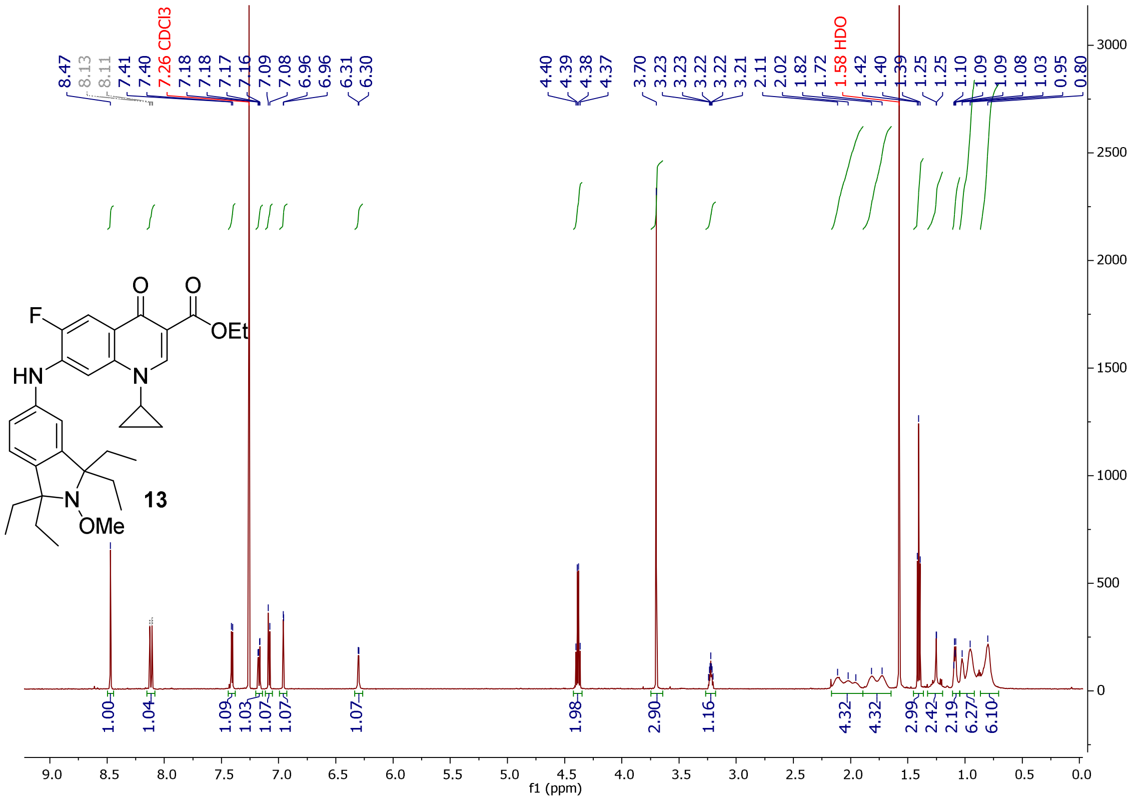

Figure S16. ^{1}H NMR ($CDCl_3$, 600 MHz) spectrum of **13**.

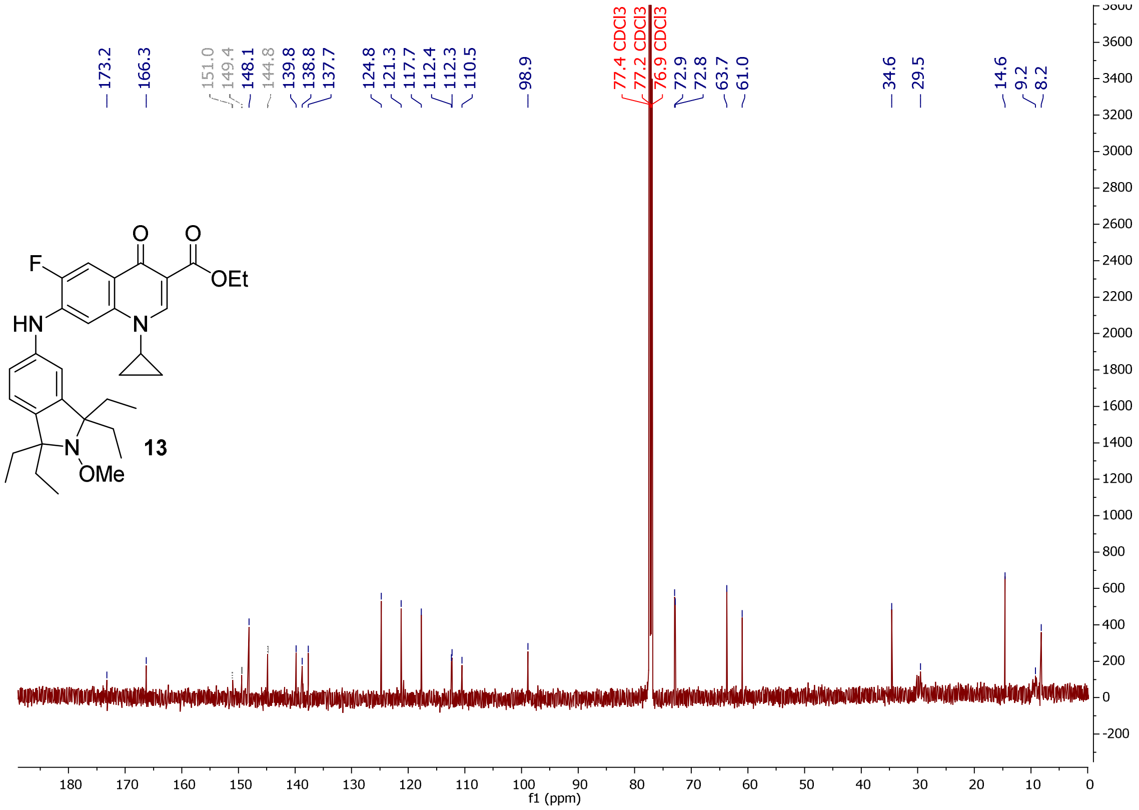

Figure S17. ^{13}C NMR ($CDCl_3$, 150 MHz) spectrum of **13**.

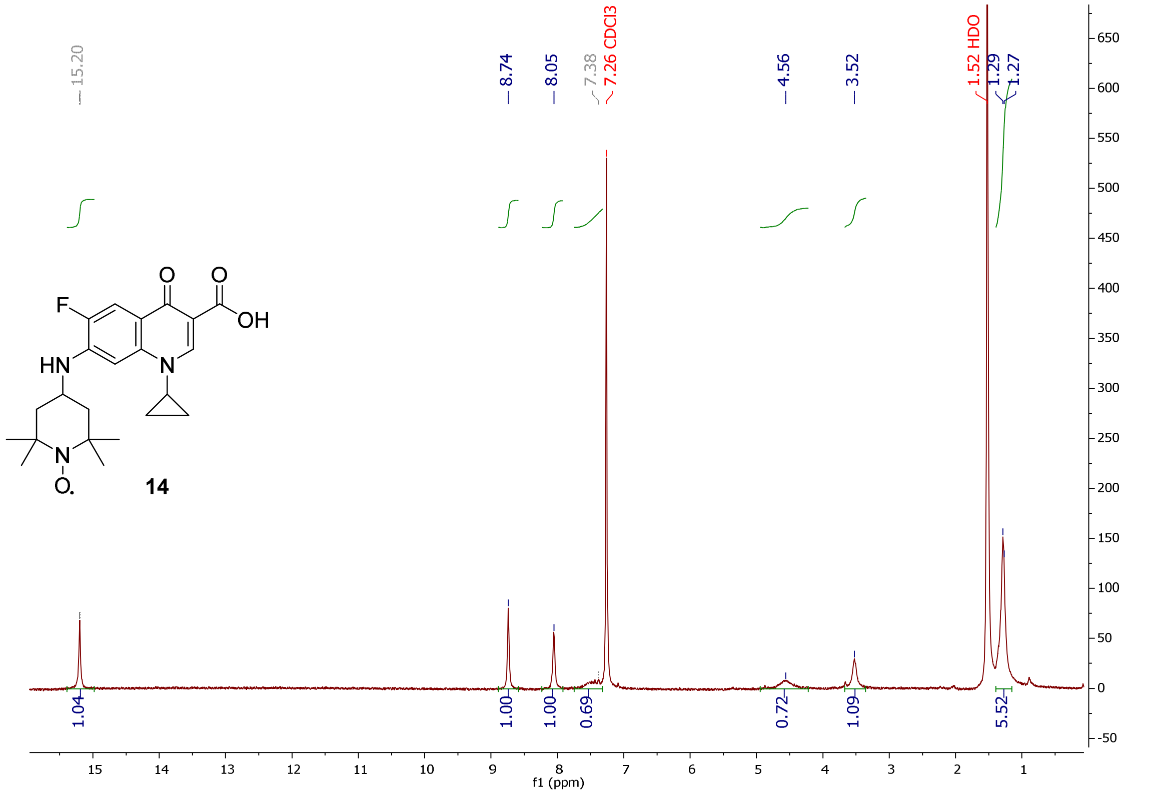

Figure S18. ^{1}H NMR ($CDCl_3$, 600 MHz) spectrum of **14**.

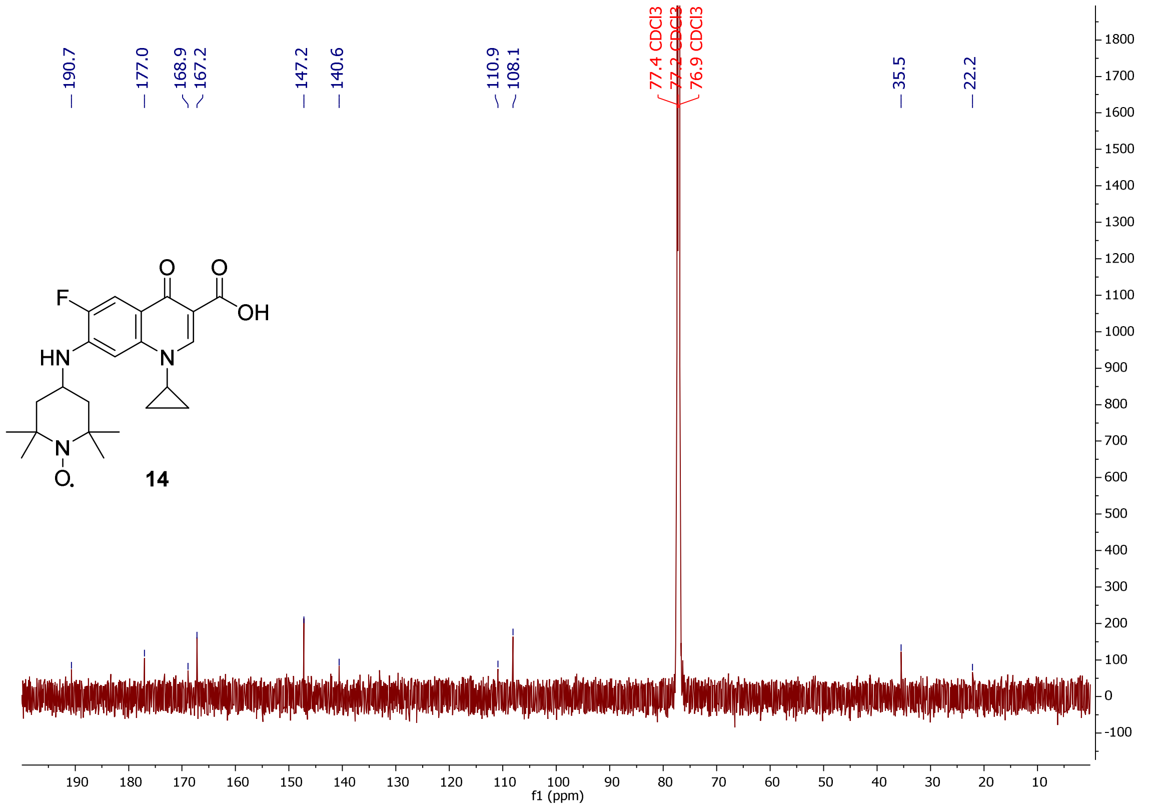

Figure S19. ^{13}C NMR ($CDCl_3$, 150 MHz) spectrum of **14**.

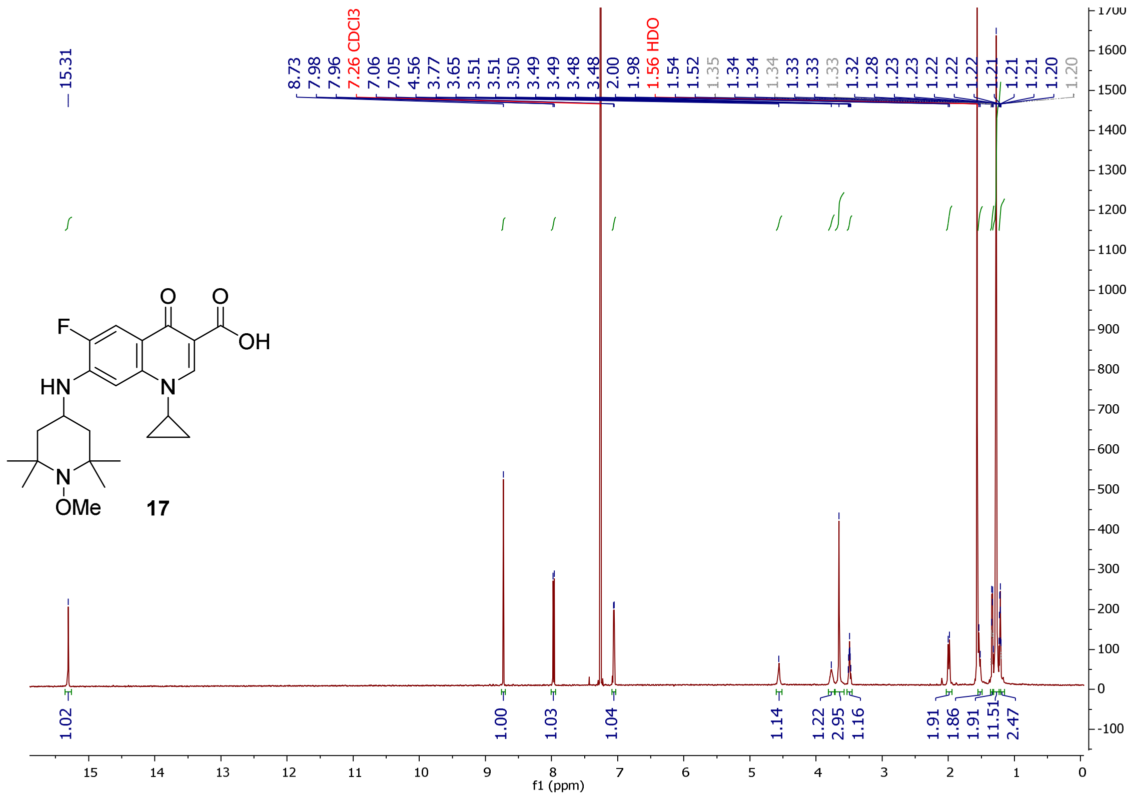

Figure S20. ^1H NMR ($CDCl_3$, 600 MHz) spectrum of **17**.

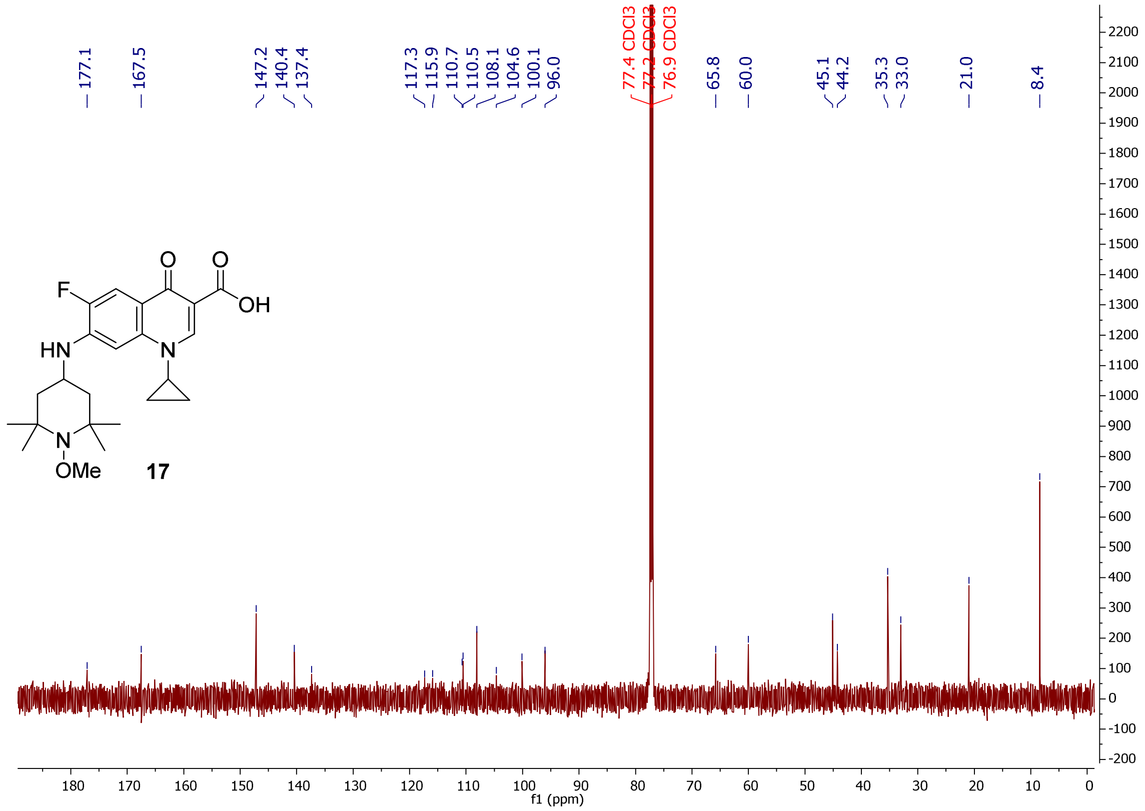

Figure S21. ^{13}C NMR ($CDCl_3$, 150 MHz) spectrum of **17**.

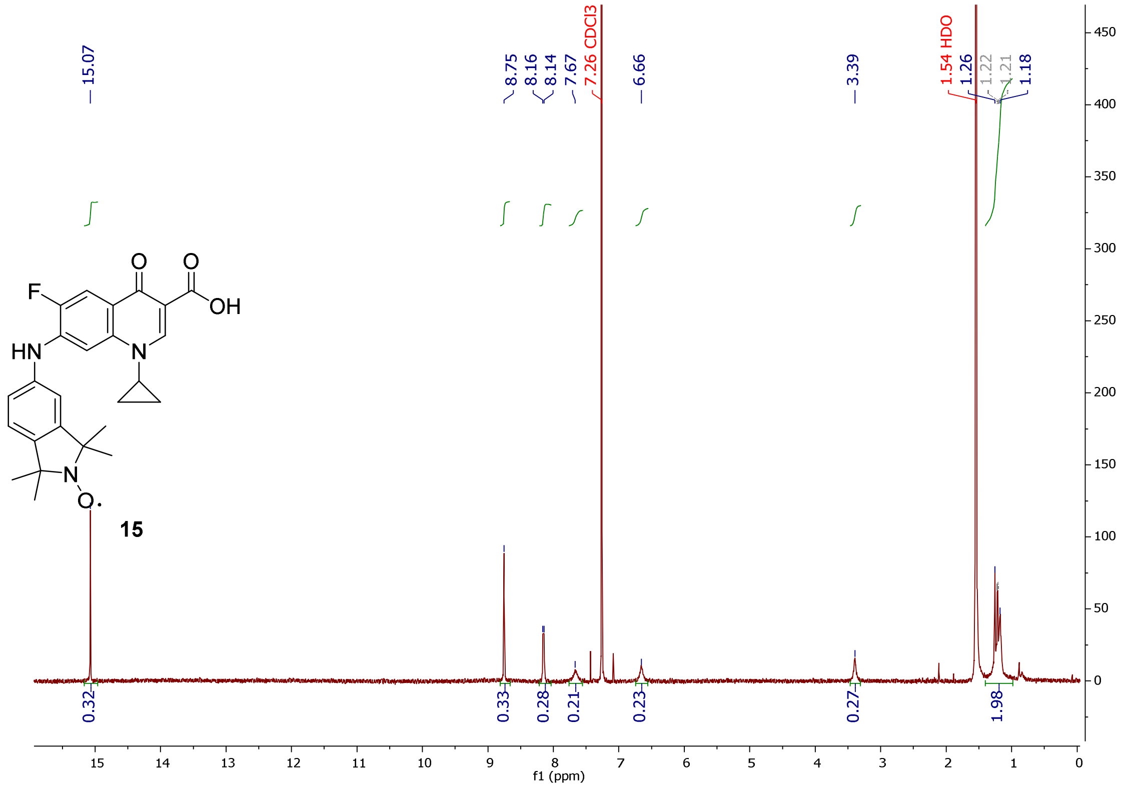

Figure S22. ^{1}H NMR ($CDCl_3$, 600 MHz) spectrum of **15**.

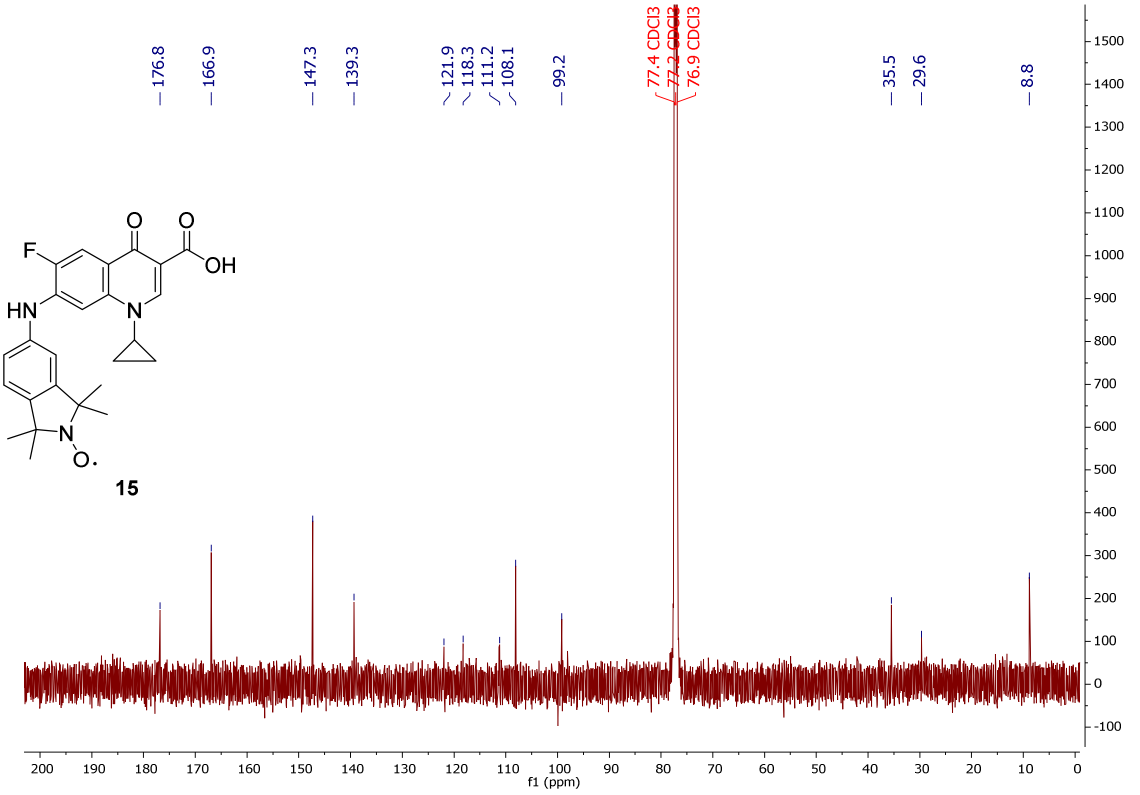

Figure S23. ^{13}C NMR ($CDCl_3$, 150 MHz) spectrum of **15**.

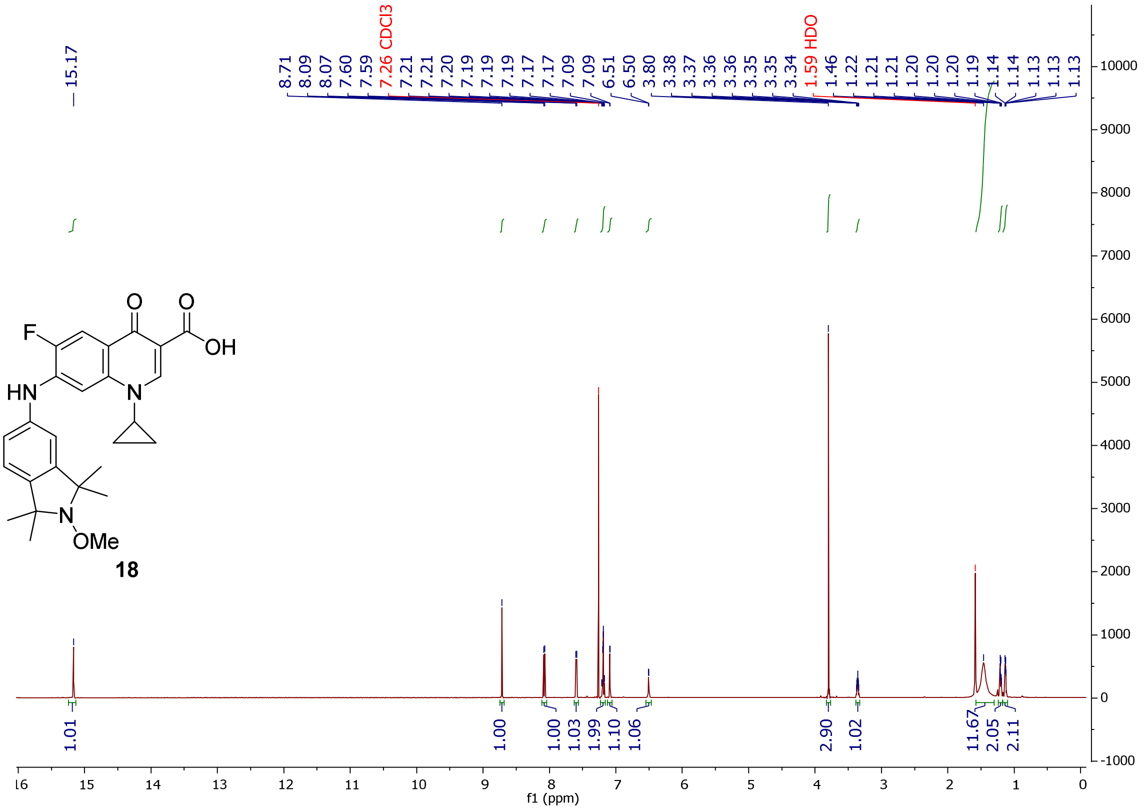

Figure S24. ^{1}H NMR ($CDCl_3$, 600 MHz) spectrum of **18**.

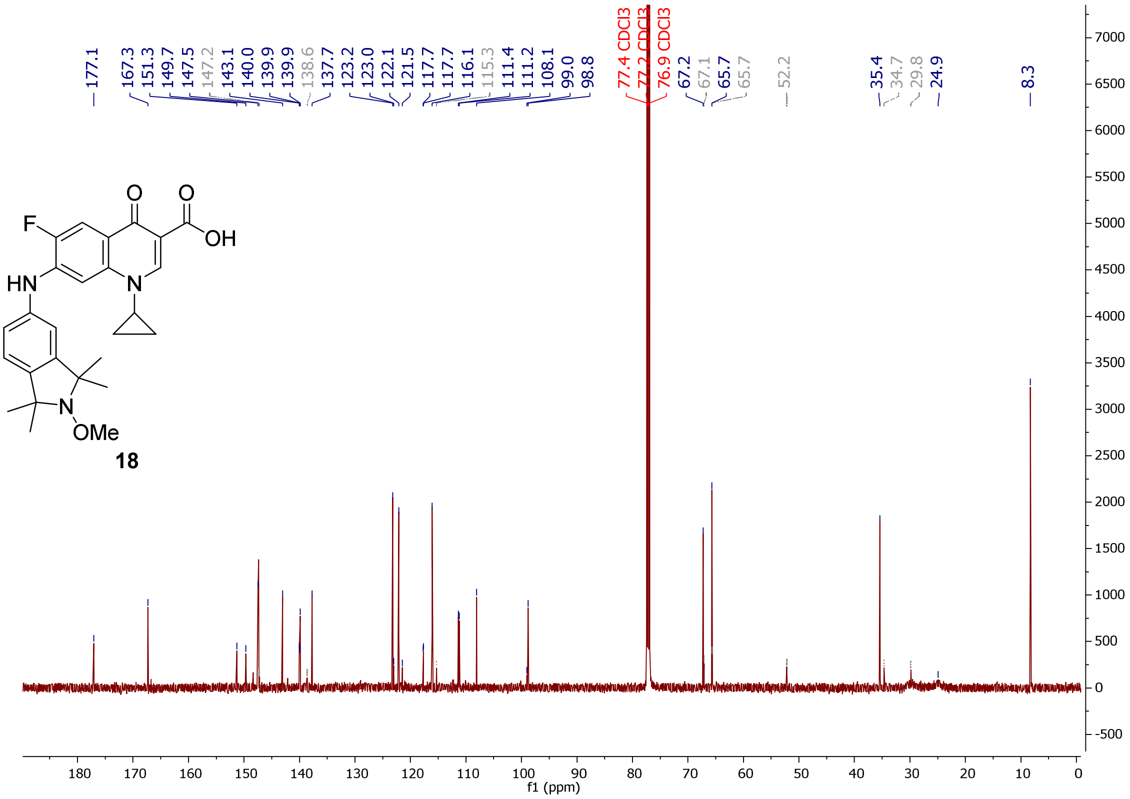

Figure S25. ^{13}C NMR ($CDCl_3$, 150 MHz) spectrum of **18**.

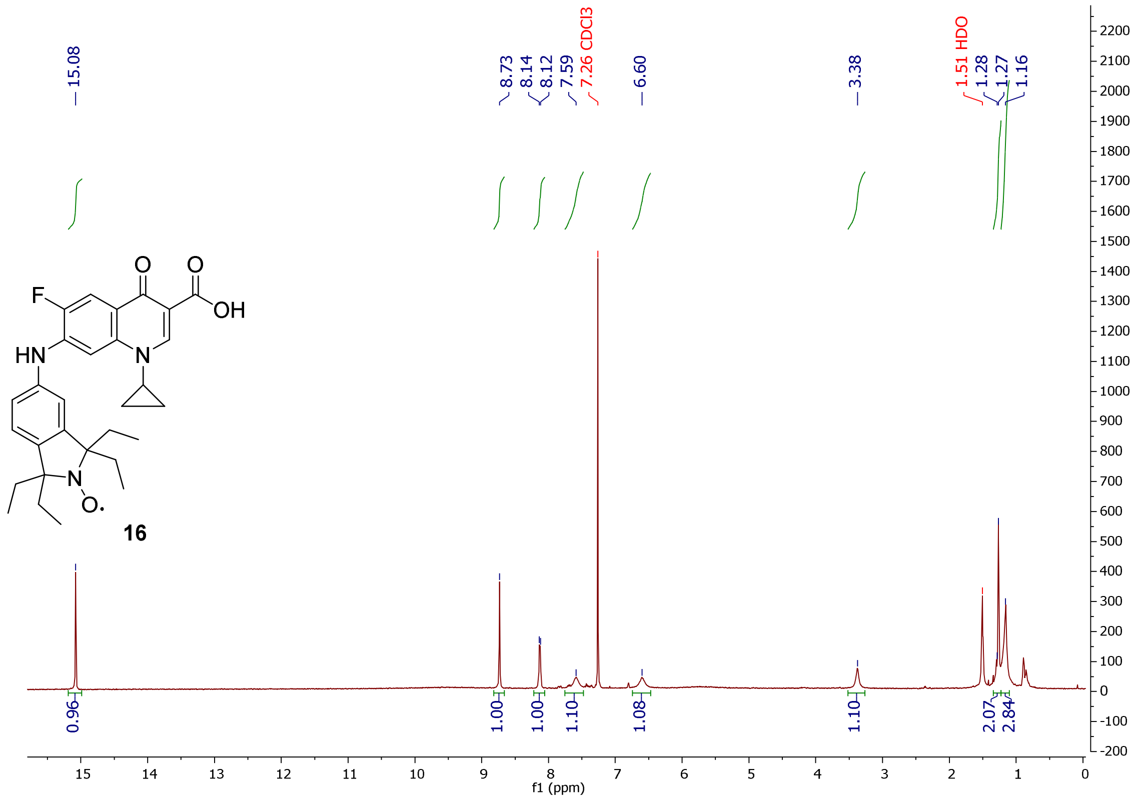

Figure S26. ^{1}H NMR ($CDCl_3$, 600 MHz) spectrum of **16**.

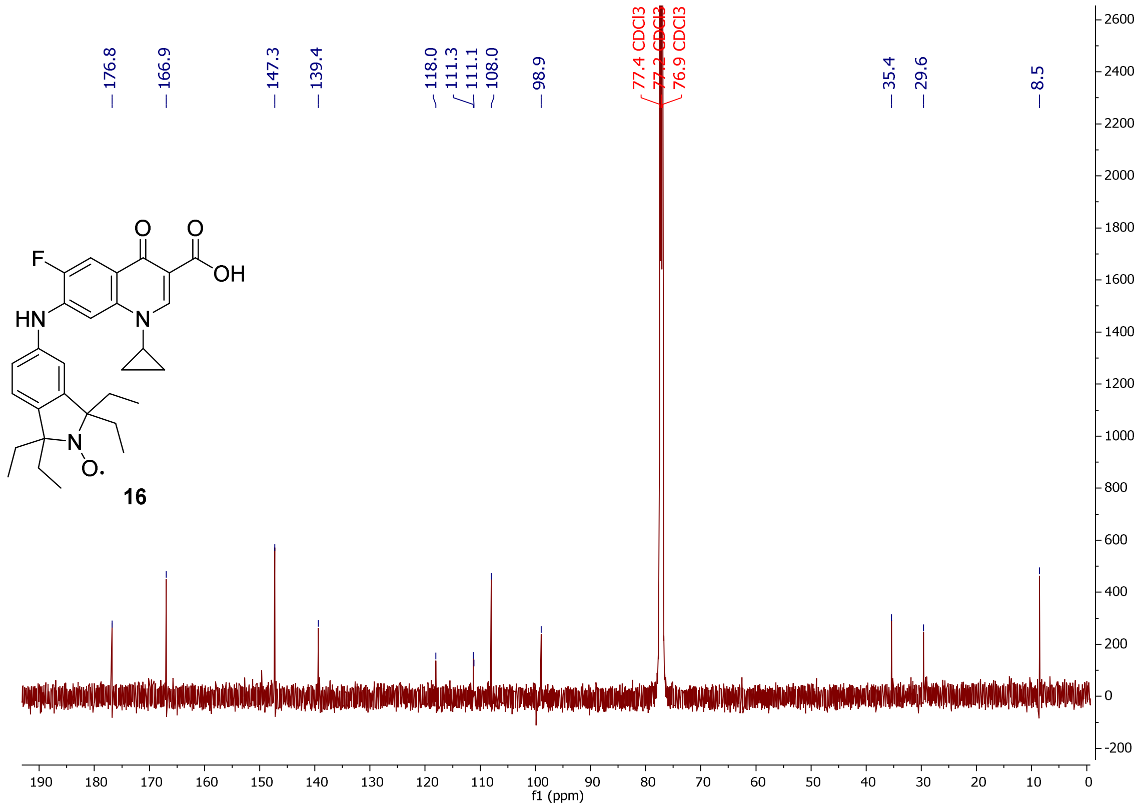

Figure S27. ^{13}C NMR ($CDCl_3$, 150 MHz) spectrum of **16**.

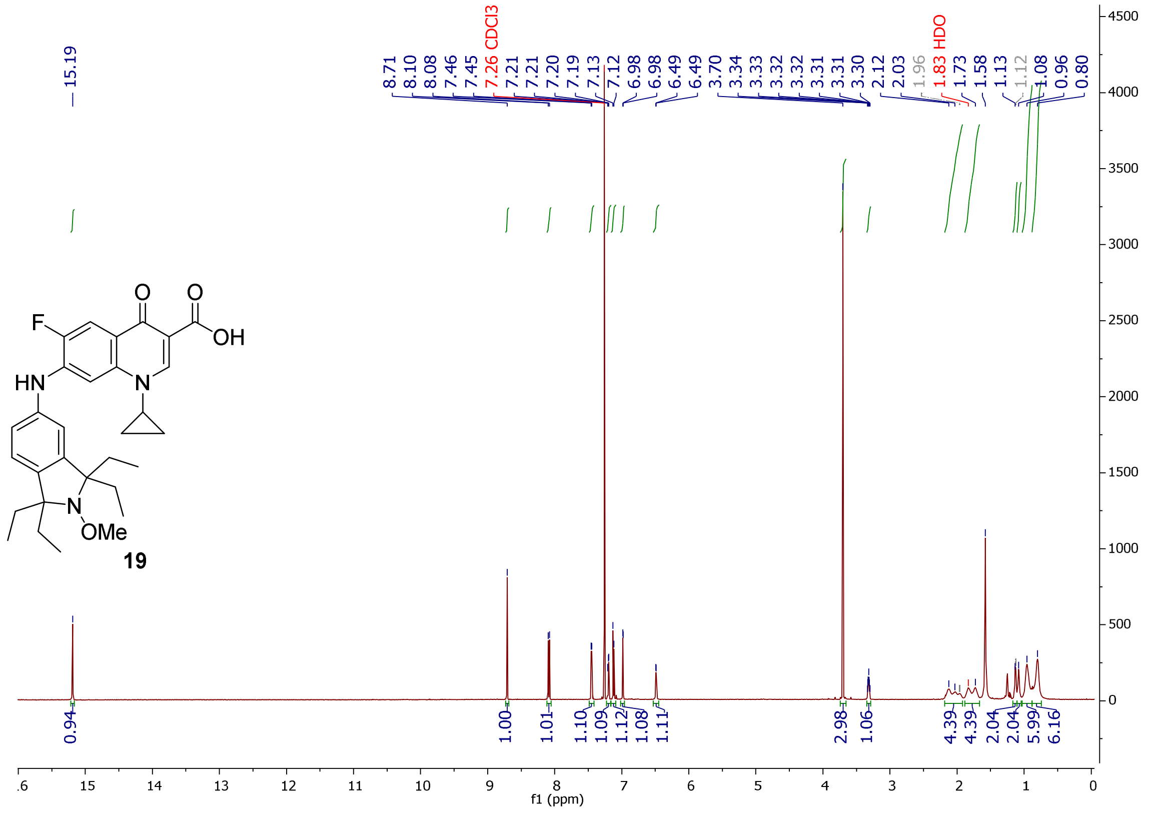

Figure S28. ^{1}H NMR ($CDCl_3$, 600 MHz) spectrum of **19**.

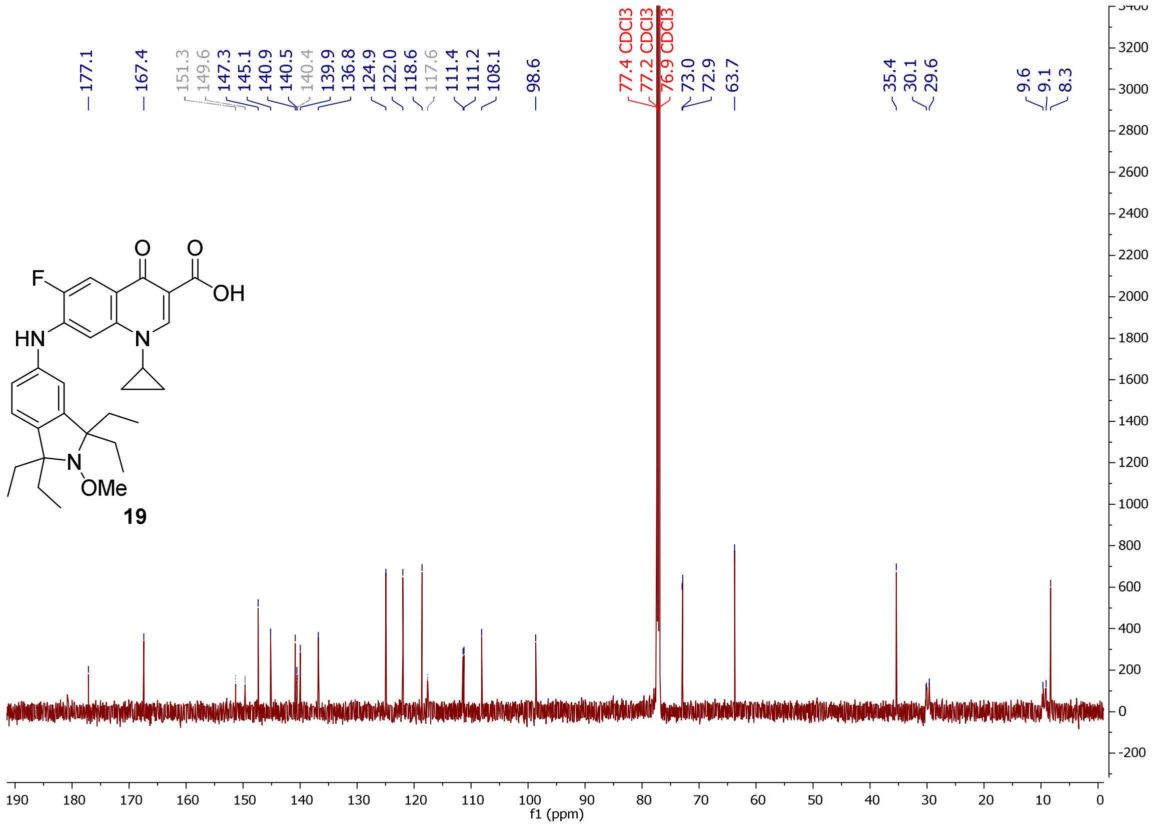

Figure S29. ^{13}C NMR ($CDCl_3$, 150 MHz) spectrum of **19**.

LCMS Chromatograms and HRMS Spectra

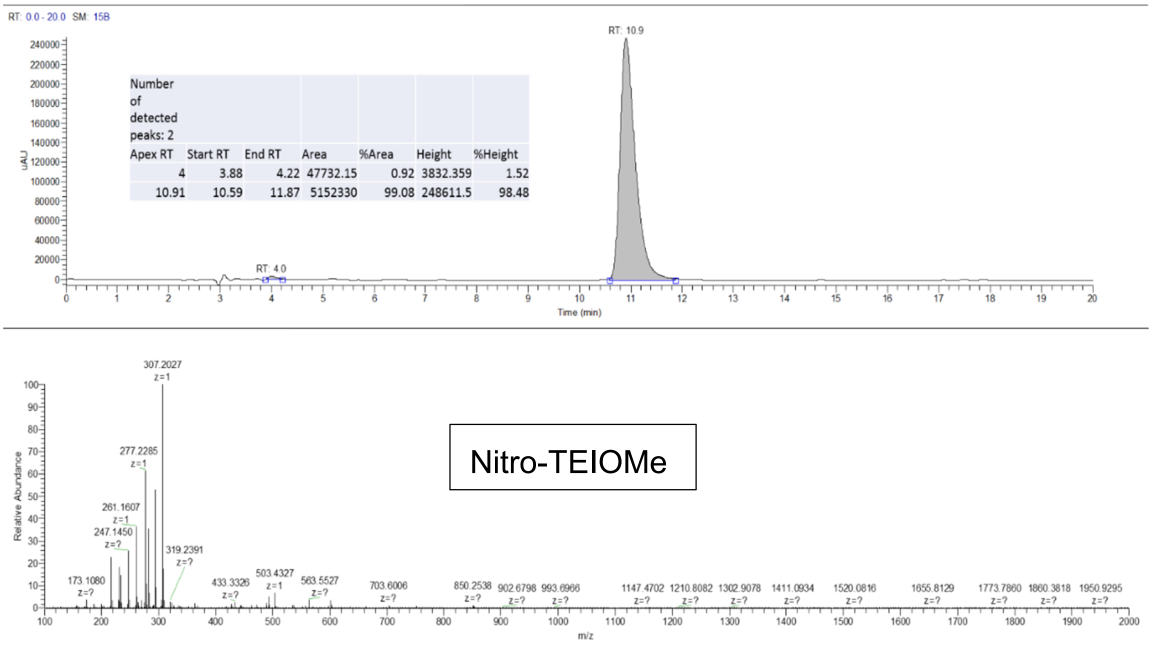

Figure S30. LCMS chromatogram and HRMS spectrum of **Nitro-TEIOMe**.

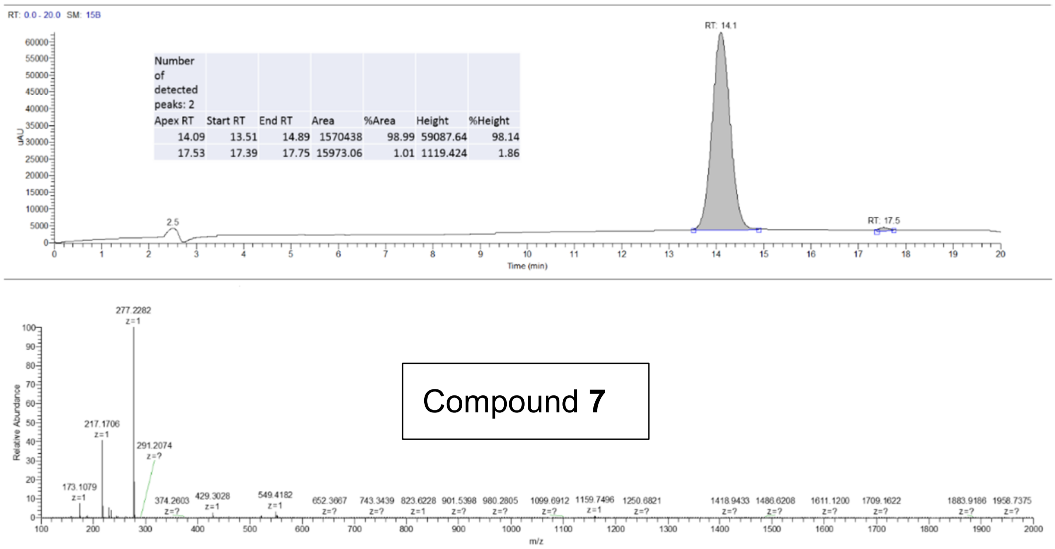

Figure S31. LCMS chromatogram and HRMS spectrum of **7**.

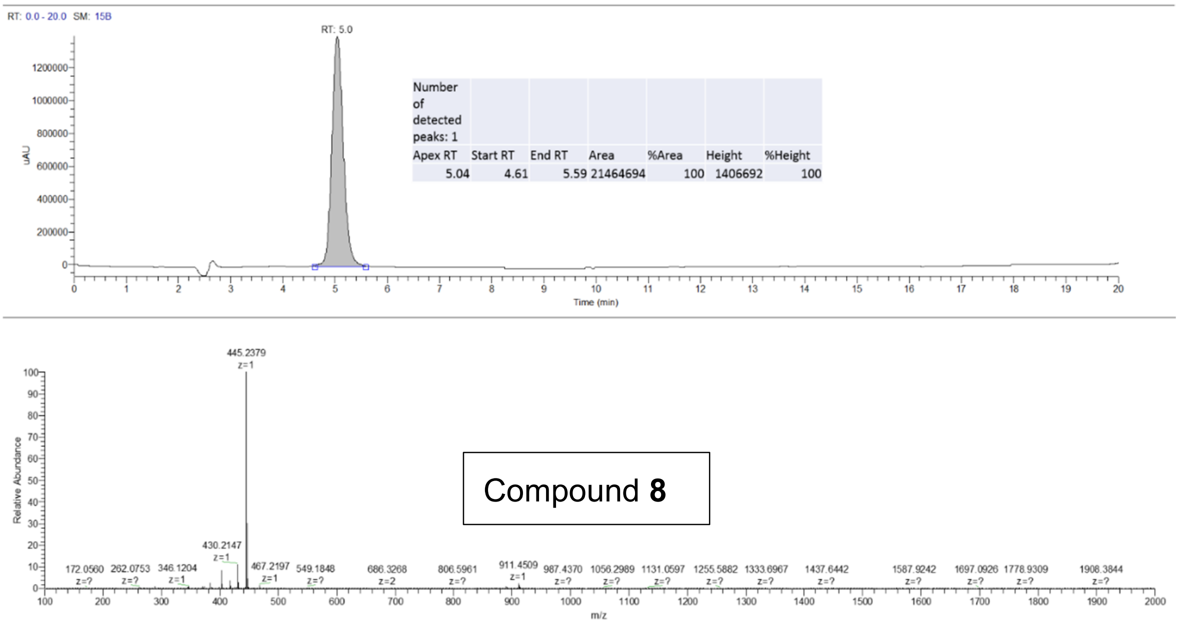

Figure S32. LCMS chromatogram and HRMS spectrum of **8**.

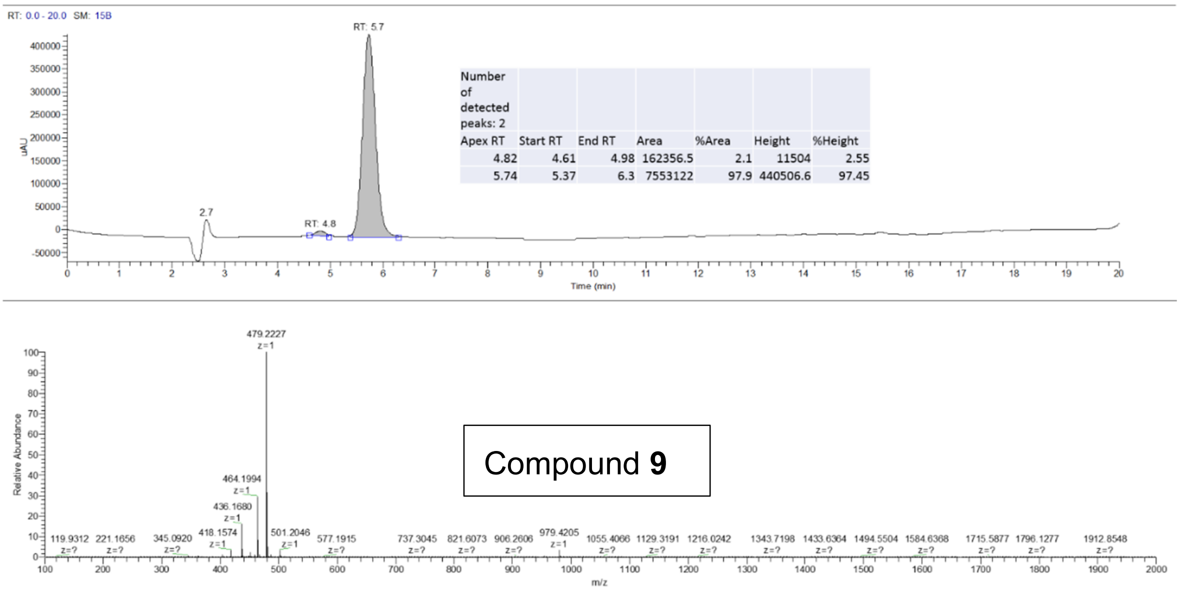

Figure S33. LCMS chromatogram and HRMS spectrum of **9**.

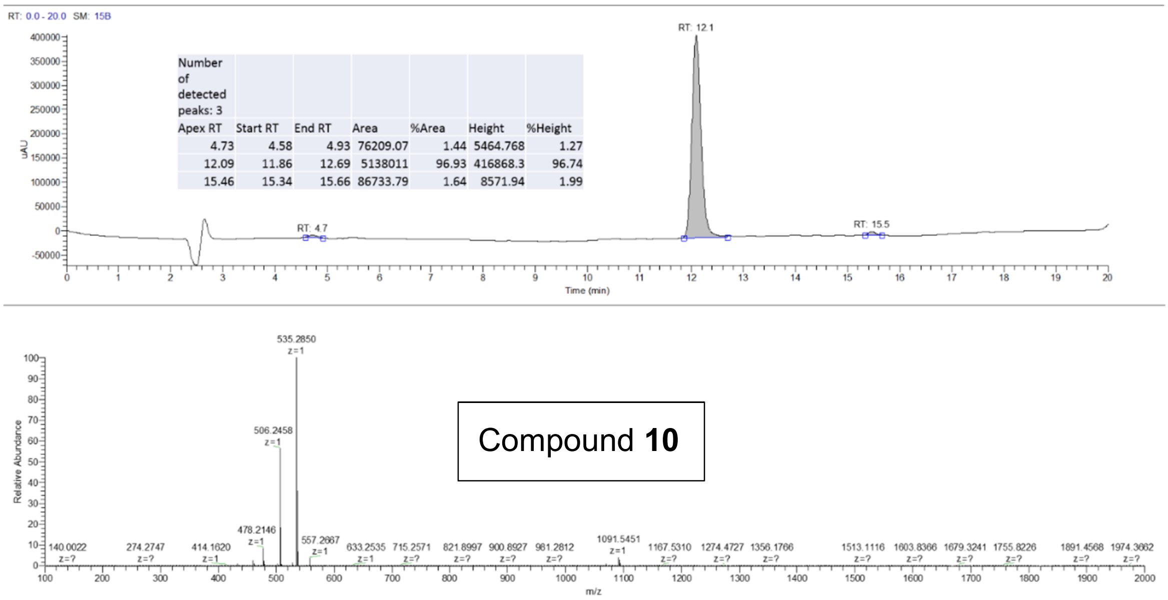

Figure S34. LCMS chromatogram and HRMS spectrum of **10**.

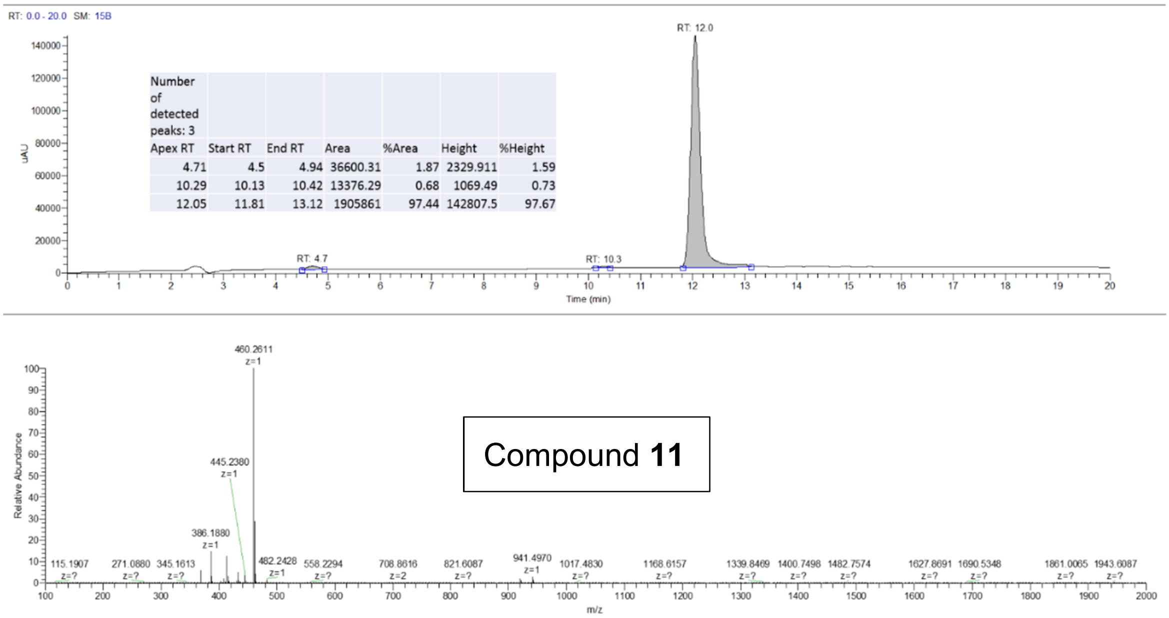

Figure S35. LCMS chromatogram and HRMS spectrum of **11**.

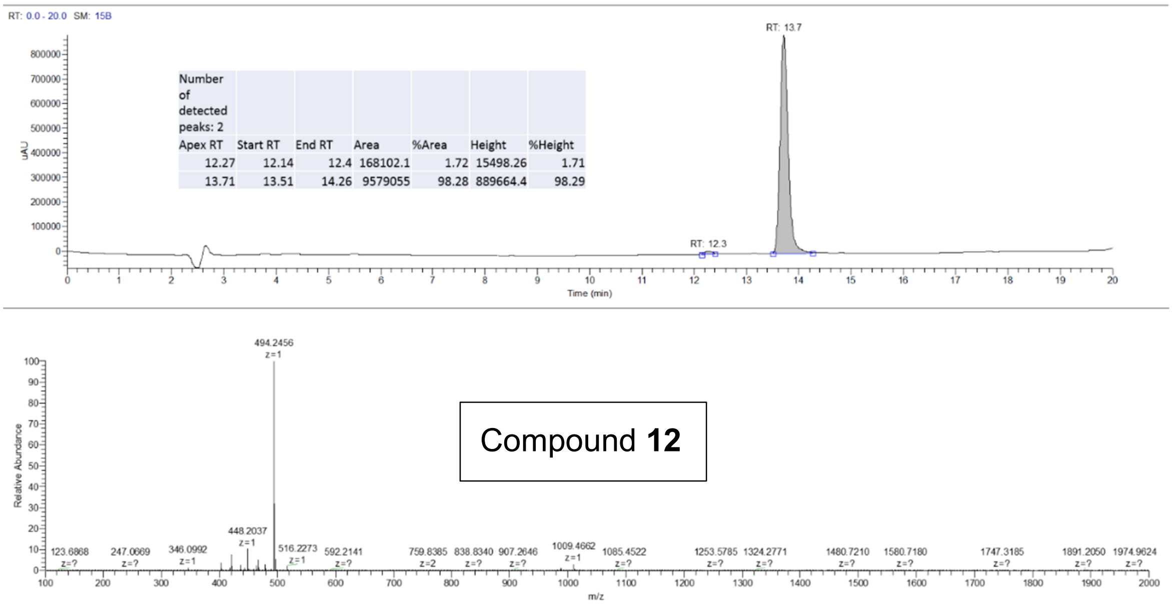

Figure S36. LCMS chromatogram and HRMS spectrum of **12**.

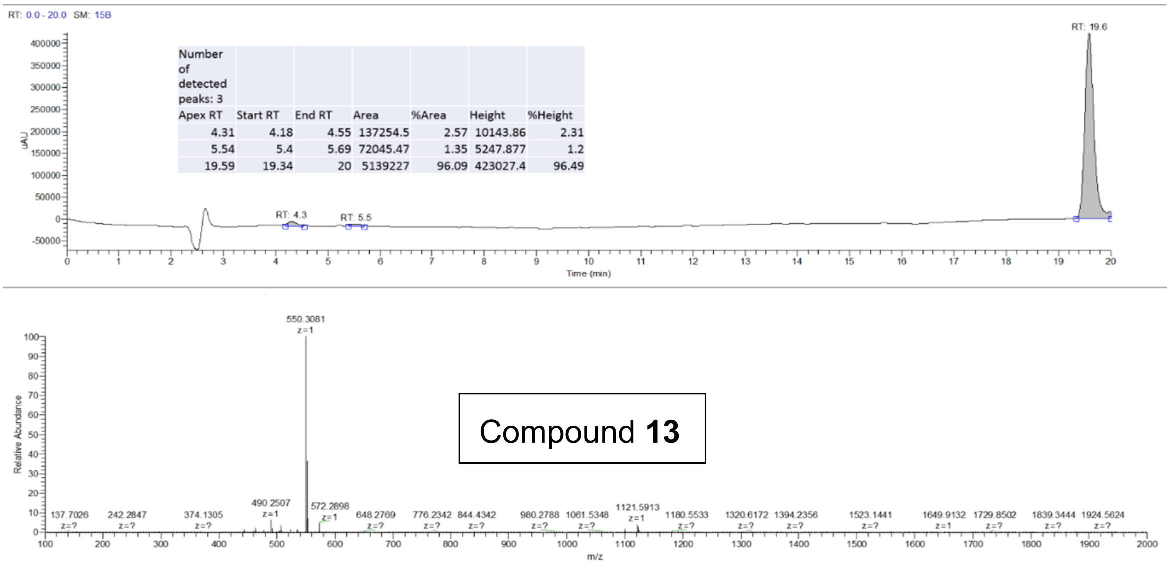

Figure 37. LCMS chromatogram and HRMS spectrum of **13**.

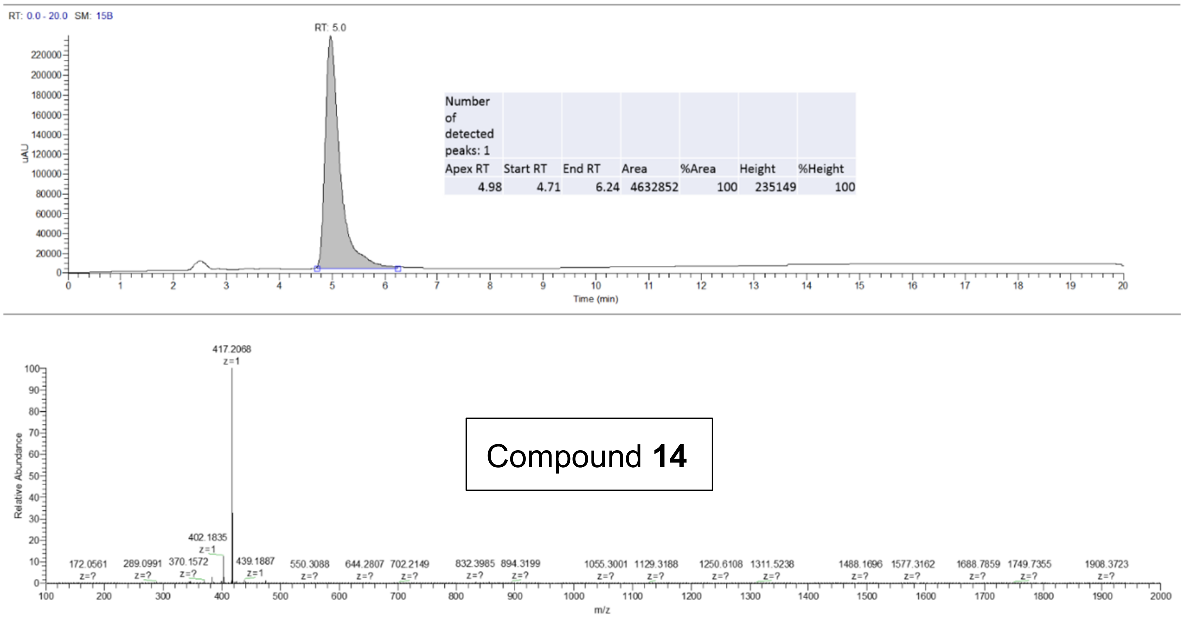

Figure S38. LCMS chromatogram and HRMS spectrum of **14**.

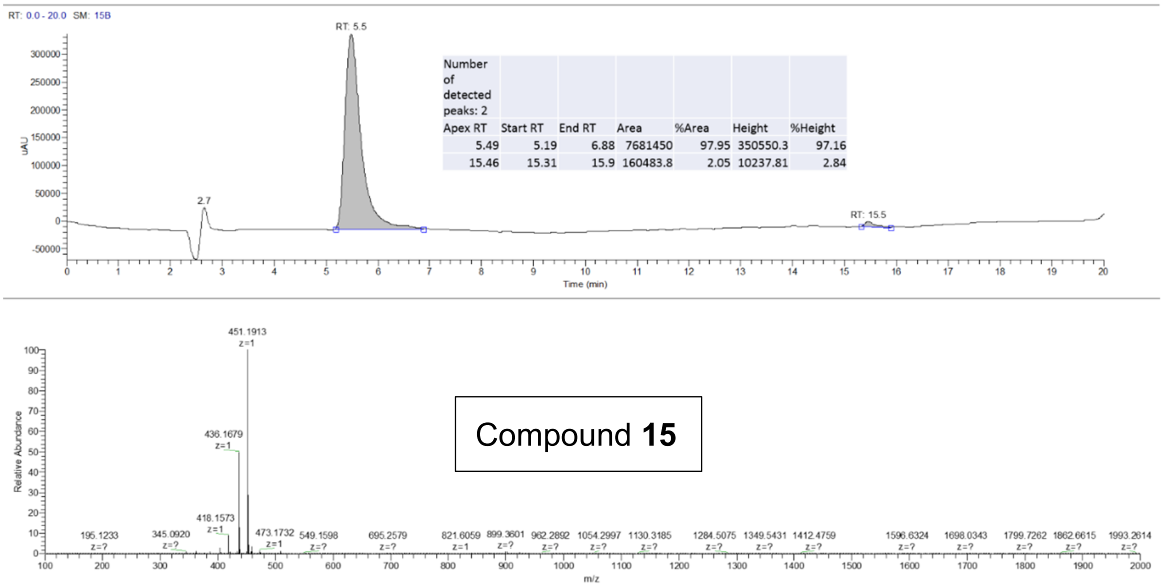

Figure S39. LCMS chromatogram and HRMS spectrum of **15**.

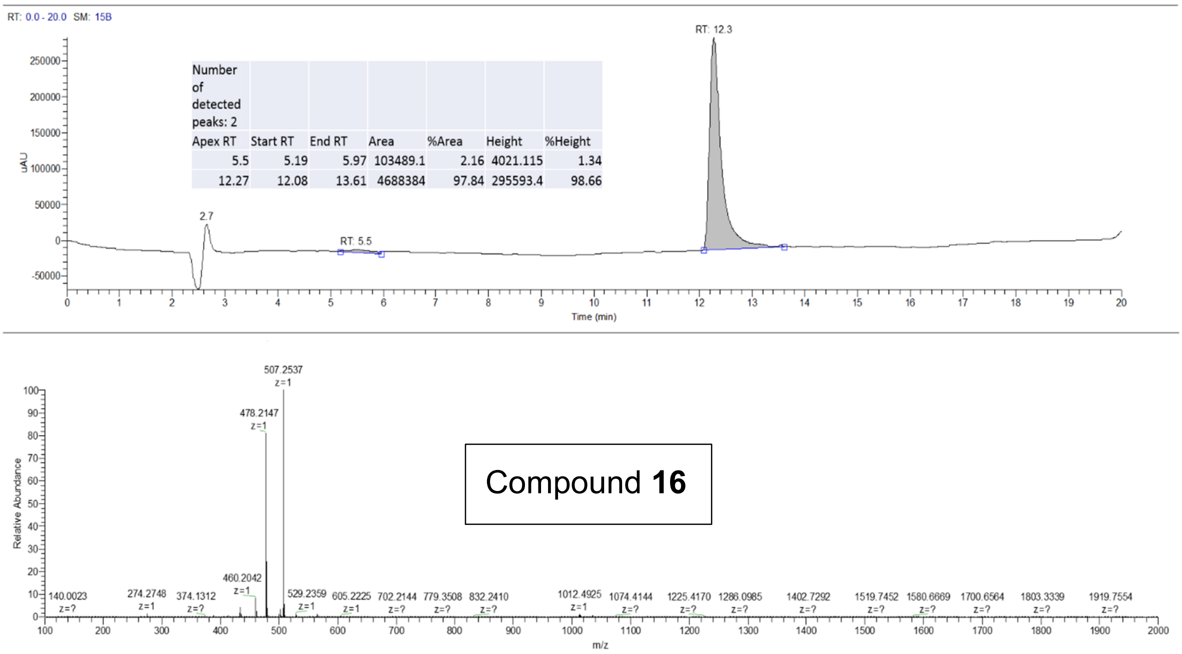

Figure S40. LCMS chromatogram and HRMS spectrum of **16**.

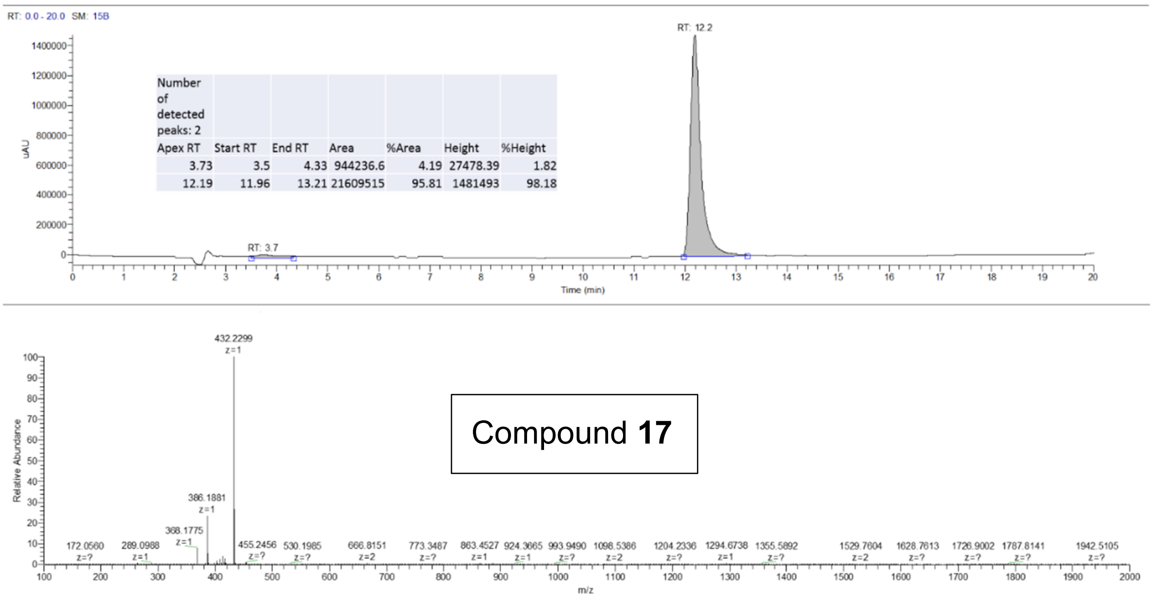

Figure S41. LCMS chromatogram and HRMS spectrum of **17**.

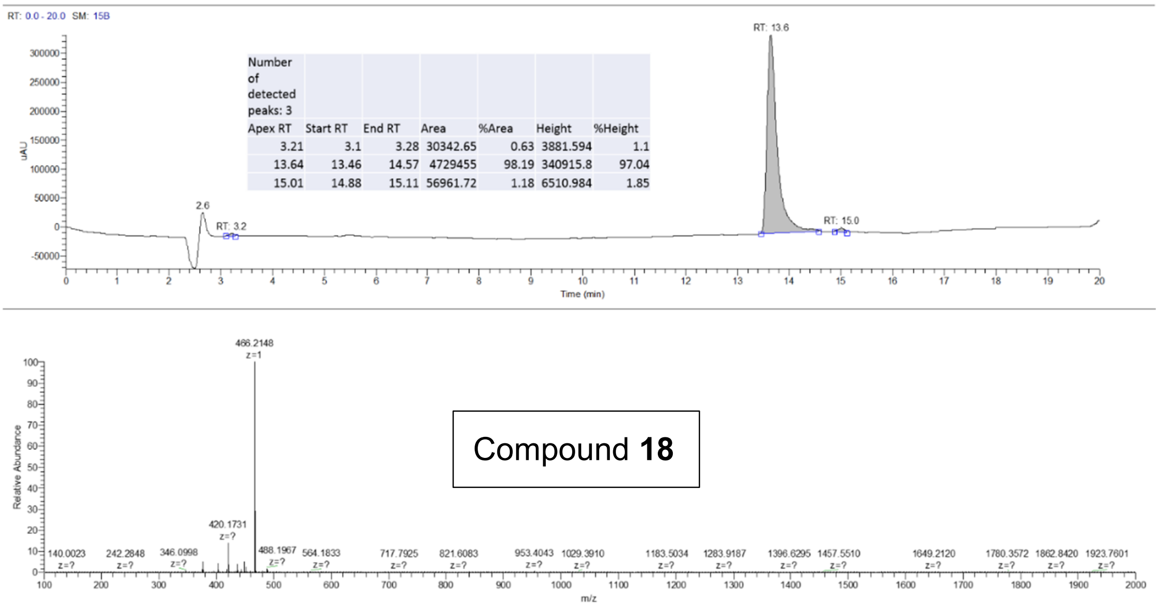

Figure S42. LCMS chromatogram and HRMS spectrum of **18**.

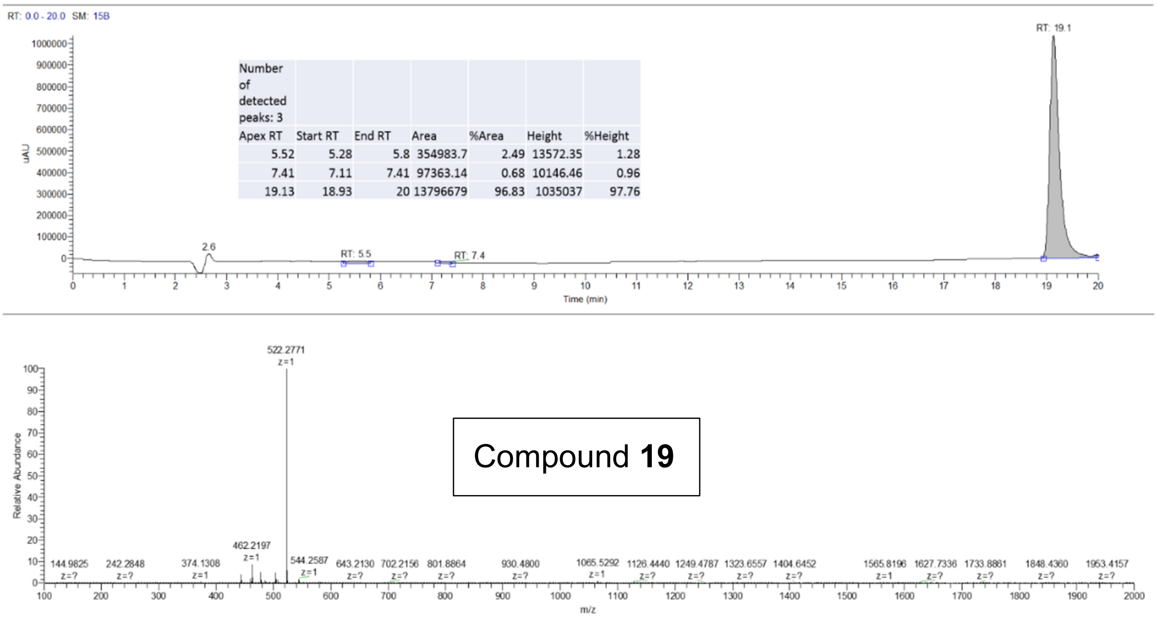

Figure S43. LCMS chromatogram and HRMS spectrum of **19**.

© 2019 by the authors. Submitted for possible open access publication under the terms and conditions of the Creative Commons Attribution (CC BY) license (http://creativecommons.org/licenses/by/4.0/).
